# SARS-CoV-2 genomic surveillance of migrants arriving to Europe through the Mediterranean routes

**DOI:** 10.7189/jogh.14.05017

**Published:** 2024-07-05

**Authors:** Fabio Tramuto, Claudia Marotta, Paola Stefanelli, Achille Cernigliaro, Carmelo Massimo Maida, Andrea Silenzi, Ulrico Angeloni, Daniela Di Naro, Giulia Randazzo, Valeria Guzzetta, Teresa Barone, Silvio Brusaferro, Santino Severoni, Gianni Rezza, Francesco Vitale, Walter Mazzucco, SAMI-Surv Collaboration, Davide Alba, Emanuele Amodio, Alessandra Casuccio, Claudio Costantino, Santo Fruscione, Palmira Immordino, Vincenzo Restivo, Alessandra Savatteri, Nadia D’Agostino, Daniele La Milia, Laura Pecoraro, Claudio Pulvirenti, Domenico Stabile, Carlo Cesari, Salvatore Zichichi, Alessandra Lo Presti, Giorgio Graziano, Salvatore Scondotto, Stefano Reale, Silvia Scibetta, Fabrizio Vitale, Chiara Barraco, Giuseppa Mistretta, Giulia Palmeri, Antonina Patrizia Rizzo, Antonino Sparaco, Annalisa Agnone, Francesco Cascio, Daniela Laura Di Quarto, Carmelo Migliorisi, Stefania D’Amato, Valentina Cucchiara, Dario Genovese, Giuseppe Friscia, Giorgia Iacolino, Vittorio Spoto, Mario Zappia

**Affiliations:** 1 Department of Health Promotion, Mother and Child Care, Internal Medicine and Medical Specialties ‘G. D’Alessandro’, University of Palermo, Italy; 2 Clinical Epidemiology Unit and Regional Reference Laboratory of Western Sicily for the Emergence of COVID-19, University Hospital ‘P. Giaccone’, Palermo, Italy; 3 General Directorate of Health Prevention, Ministry of Health, Rome, Italy; 4 National Institute of Health (Istituto Superiore di Sanità), Rome, Italy; 5 Regional Health Authority of Sicily, Palermo, Italy; 6 Department of Laboratory Diagnostics, Local Health Unit of Palermo, Palermo, Italy; 7 University of Udine, Udine, Italy; 8 Health and Migration Programme (PHM), World Health Organization, Geneva, Switzerland; 9 Vita – Salute San Raffaele University, Milan, Italy; 10 Division of Biostatistics & Epidemiology Research, Cincinnati Children’s Hospital Medical Center, Cincinnati, USA

## Abstract

**Background:**

The implementation genomic-based surveillance on emerging severe acute respiratory syndrome coronavirus 2 (SARS-CoV-2) variants in low-income countries, which have inadequate molecular and sequencing capabilities and limited vaccine storage, represents a challenge for public health. To date, there is little evidence on molecular investigations of SARS-CoV-2 variants in areas where they might emerge. We report the findings of an experimental SARS-CoV-2 molecular surveillance programme for migrants, refugees, and asylum seekers arriving to Europe via Italy through the Mediterranean Sea.

**Methods:**

We descriptively analysed data on migrants collected at entry points in Sicily from February 2021 to May 2022. These entry points are integrated with a network of laboratories fully equipped for molecular analyses, which performed next-generation sequencing and used Nextclade and the Pangolin coronavirus disease 2019 (COVID-19) tools for clade/lineage assignment.

**Results:**

We obtained 472 full-length SARS-CoV-2 sequences and identified 12 unique clades belonging to 31 different lineages. The delta variant accounted for 43.6% of all genomes, followed by clades 21D (Eta) and 20A (25.4% and 11.4%, respectively). Notably, some of the identified lineages (A.23.1, A.27, and A.29) predicted their introduction into the migration area. The mutation analysis allowed us to identify 617 different amino acid substitutions, 156 amino acid deletions, 7 stop codons, and 6 amino acid insertions. Lastly, we highlighted the geographical distribution patterns of some mutational profiles occurring in the migrants’ countries of origin.

**Conclusions:**

Genome-based molecular surveillance dedicated to migrant populations from low-resource areas may be useful for forecasting new epidemiological scenarios related to SARS-CoV-2 variants or other emerging pathogens, as well as for informing the updating of vaccination strategies.

The Central Mediterranean migration route to Europe is one of the most significant and dangerous routes in the world, and it continues to be a major humanitarian concern. It is characterised by a mixed migration flow, with diverse groups of people from Africa, the Middle East, and Asia attempting to reach European shores in search of safety, economic opportunities, and a better life [1-3]. Since the early 2000s, thousands of migrants, refugees, and asylum seekers have been crossing the Central Mediterranean Sea route every year, either via Tunisia or Libya [1]. They often do so by travelling on makeshift or illegal boats; in cases of disasters, they have been rescued in the sea or land in Italy [1]. Within this context, Sicily and its smaller islands represent the main entrance to Europe from Northern African borders [4,5].

Conflicts, economic inequalities, climate change, and political instability in the countries of origin have impacted Mediterranean migration trends. For example, the 2011 Arab Spring uprisings led to an upsurge in migrants and refugees, mainly from Syria, Libya, and other North African and Middle Eastern countries [6]. These movements have fluctuated over the years due to European policy changes, the stability of countries along the route, and international agreements to limit the flow. The coronavirus 2019 (COVID-19) pandemic introduced additional complexities to these migration patterns [7]. While the pandemic and the resultant global economic downturn have intensified the root drivers of migration, such as unemployment, poverty, and political instability in countries of origin, the movement restrictions imposed to mitigate virus transmission temporarily reduced the number of migrants able to undertake the journey. Thus, despite unprecedented national and international restrictions to mitigate the spread of the virus, including ‘lockdowns’ and travel bans [8,9], migration movement decreased, but never ceased [10].

After a migrant arrives in Italy, the cross-border health care services of the Italian Ministry of Health (*Ufficio di Sanità Marittima, Aerea e di Frontiera *(*USMAF*)) initiates the disembarkation process, while local health authorities provide full medical screening and support to each migrant in order to detect and prevent the entry of communicable diseases of potential public health concern, ensure public health surveillance, and address any health care needs by facilitating access to health care services.

The management of large-scale migration, both for the migrants themselves and for the host countries, was a significant public health challenge during the pandemic. Overcrowding was likewise a major issue, both in transit on unsafe vessels and within host centres, where maintaining physical distancing or proper isolation was unfeasible, creating conditions that facilitated respiratory disease transmission, including COVID-19. Migrants’ limited access to health care, both in transit and upon arrival, impeded the early detection and treatment of infectious diseases, potentially worsening health outcomes and facilitating broader transmission. Vulnerable populations, including children, pregnant women, the elderly, and individuals with pre-existing health conditions, were at higher risk in these circumstances.

Since the combination of an uncontrolled spreading of severe acute respiratory syndrome coronavirus 2 (SARS-CoV-2) and the limited availability of vaccines may have facilitated the selection pressure with the emergence of new variants of concern (VOCs) [11], full genome sequencing of SARS-CoV-2 virus quickly gained a key role in the global public health approach to combatting the virus [12]. Thus, a specific SARS-CoV-2 surveillance programme was established for migrants arriving in Europe via Italy, which was later upgraded at the Sicilian borders with genomics investigation to improve the knowledge of SARS-CoV-2 transmission dynamic [13]. To this end, the country implemented an experimental SARS-CoV-2 interinstitutional surveillance programme dedicated to migrants, refugees, and asylum seekers arriving to Italy through the Mediterranean Sea, supported by a network of laboratories fully equipped for molecular and full genome sequencing analyses [14].

Here we report on the findings of this programme and discuss their implications for public health, including the extension of this COVID-19 experimental border surveillance model on a global scale and to other diseases related to emergent or re-emerging pathogens.

## METHODS

### Study population and design

We performed a descriptive study on a sample of migrants arriving through a mixed migration flow in the Mediterranean to Sicily (Italy) from February 2021 to May 2022, during the state of pandemic emergency which was extended to December 2022 in the Sicilian region (**Box 1**) [16,17]. We reported our findings according to STROBE guidelines [18].

Box 1 **Study population and definitions.**

The Central Mediterranean route, with Italy as the primary entry point into Europe, experiences a mixed migration flow. This is a multifaceted cross-border population movement involving refugees, asylum seekers, economic migrants, and other types of migrants, as opposed to other migration movements that solely comprise one category of migrants. Driven by various factors, individuals involved in mixed migration have different legal statuses, while sharing the same route and means of travel, often engaging in irregular travel and relying on human smugglers.

In line with our study aim, we refer to each component of the whole population with the generic term ‘migrant,’ bearing in mind that this includes different types of migrants as per the below-listed definitions from the Glossary on Migration provided by the International Organization for Migration in 2018 [15]:

**International migrants: any person who changes his or her country of usual residence.**
− Refugees: any person who meets the eligibility criteria under an applicable definition of refugee, as provided for in international or regional refugee instruments, under the mandate of the United Nations High Commissioner for Refugees (UNHCR) or in national legislation. Refugees are persons outside their country of origin who need international protection because they fear persecution or a serious threat to their life, physical integrity or freedom in their country of origin as a result of persecution, armed conflict, violence or serious public disorder.
− Asylum seeker: an individual who seeks international protection. They are people whose claim has not been finally decided on by the country in which they have submitted it. Not every asylum seeker will ultimately be recognised as a refugee, but every recognised refugee is initially an asylum seeker.

Migrants received medical screening upon their arrival in Sicily, with their nasopharyngeal swabs regularly tested for SARS-CoV-2. The local staff also collected demographic data, migration routes, and essential clinical data suggestive of COVID-19. Moreover, according to national COVID-19 regulations, all migrants were quarantined in dedicated reception facilities (camps or reconverted cruise ships) [14]; each subject testing positive was isolated, after which contact tracing was conducted. All confirmed positive cases were reported to the COVID-19 integrated surveillance system under the supervision of the Italian National Institute of Health (*Istituto Superiore di Sanità*) [19].

The SARS-CoV-2 positive samples were then shipped regularly to the hub laboratory, where full genome sequencing analyses were performed until one week from the delivery (on average). We summarised the participants’ demographic and clinical characteristics, as well as the distribution of the identified SARS-CoV-2 clades and lineages, as frequencies and percentages or median values with interquartile ranges (IQRs), as appropriate. We then accessed the Global Initiative on Sharing All Influenza Data (GISAID) database [20] to select genomes belonging to the lineages identified among migrants, according to date of first genome detections.

### SARS-CoV-2 detection, genome sequencing and clade/lineage assignment

Viral RNA was extracted from nasopharyngeal specimens using QIAamp Viral RNA extraction kit (QIAGEN) and then tested for SARS-CoV-2 detection using singleplex one-step retro-transcription real-time polymerase chain reaction (rt-RT-PCR) assays targeting the N gene of SARS-CoV-2 [21].

We then selected samples with appropriate viral concentration (rt-RT-PCR cycle threshold (Ct) values ≤32) for next-generation sequencing on an Ion GeneStudio S5 System (Applied Biosystems) and generated virus genomes by using a multiplex approach using the Ion Ampliseq SARS-CoV-2 Research Panel.

We designated SARS-CoV-2 lineages using the dynamic nomenclature system proposed by Rambaud et al. [22]; here we refer to them as ‘Pango lineages.’ We also assigned SARS-CoV-2 genomes to ‘Nextstrain clades’ using the Nextclade tool, version 3.7.4. [23]. Pangolin is the most widely used classification tool for automatic assignment of lineages, while Nextclade has been used to assign Pango lineages to SARS-CoV-2 sequences worldwide [24]. All SARS-CoV-2 genomes included in the study were submitted to the GISAID repository (Table S2 in the **Online Supplementary Document**).

### Ethics statement

We carried out this study in full compliance with the European Community rules on data protection and the Declaration of Helsinki. We obtained formal approval (number 7/2020) from the Palermo 1 ethics committee at the Palermo University Hospital, Italy. Individual data were collected in an emergency operative setting under the Italian laws regulating the COVID-19 pandemic surveillance, so informed consent was waived. We presented the data in fully anonymised, aggregate form per the European General Data Protection Regulation.

## RESULTS

**Figure 1:**
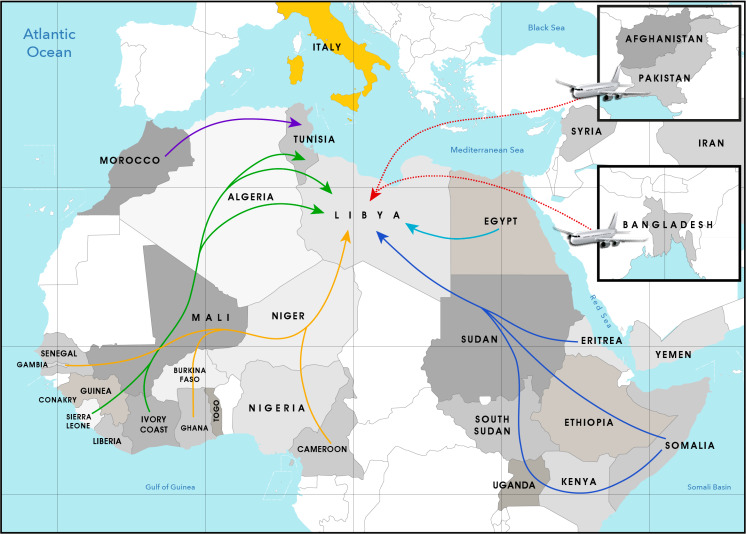
Geographic map showing the main routes documented among the convenience sample of migrants who arrived in Italy by the Mediterranean Sea (February 2021 to May 2022).

We included 704 SARS-CoV-2 positive migrants in the study, most of whom were males (n = 650, 92.3%). Their median age was 22.0 years (IQR = 9.0); 672 (95.5%) had a traceable origin (**Figure 1**, **Table 1****;** Table S1 in the **Online Supplementary Document**): almost half were from countries within Mediterranean Africa (46.0%; 188 were from Tunisia, 86 from Egypt, 30 from Morocco, and 1 from Libya), followed by sub-Saharan Africa (22.5%; 33 were from Ivory coast, 22 from Sudan, 19 from Guinea Conakry, 14 from Mali, 14 from Burkina Faso, 12 from Cameroon, 9 from Ghana, 7 from Gambia, 6 from Nigeria, 5 from Senegal, 3 from Sierra Leone, 3 from Guinea, 2 from Togo, and 1 from Niger), Southeast Asia (19.1%; 127 were from Bangladesh and 1 from Indonesia), the Middle East (6.5%; 7 from Afghanistan, 19 from Syria, 13 from Iran, 5 from Pakistan, and 1 from Yemen) and the Horn of Africa (5.9%; 20 were from Eritrea, 11 from Somalia, 8 from Ethiopia, and 1 from Kenya), respectively. Groups coming from Afghanistan, Bangladesh, and Pakistan arrived from Libya by way of airplanes (**Figure 1**).

**Table 1:** Demographics and clinical characteristics of the sample of 704 migrants in study*

Sex	
Female	54 (7.7)
Male	650 (92.3)
**Age in years, median (IQR)**	22.0 (9.0)
**Age groups in years**	
≤5	11 (1.6)
6-13	11 (1.6)
14-17	131 (18.6)
18-22	229 (32.6)
23-28	172 (24.5)
29-49	143 (20.3)
≥50	6 (0.8)
Total	703 (100)
**Geographic macro-areas of origin**	
Mediterranean Africa	309 (46.0)
Sub-Saharan Africa	151 (22.5)
Southeast Asia	128 (19.1)
Middle East	44 (6.5)
Horn of Africa	40 (5.9)
Total	672 (100)
**Symptoms suggestive of COVID-19**	
None	585 (93.6)
Mild symptoms	39 (6.2)
Severe symptoms	1 (0.2)
Total	625 (100)

IQR – interquartile range

*Values presented as n (%) unless specified otherwise.

Among migrants with available data on their clinical status, 93.6% were asymptomatic, 6.2% showed mild symptoms suggestive of COVID-19, and only one case (0.2%) had a severe clinical presentation (**Table 1**).

**Figure 2:**
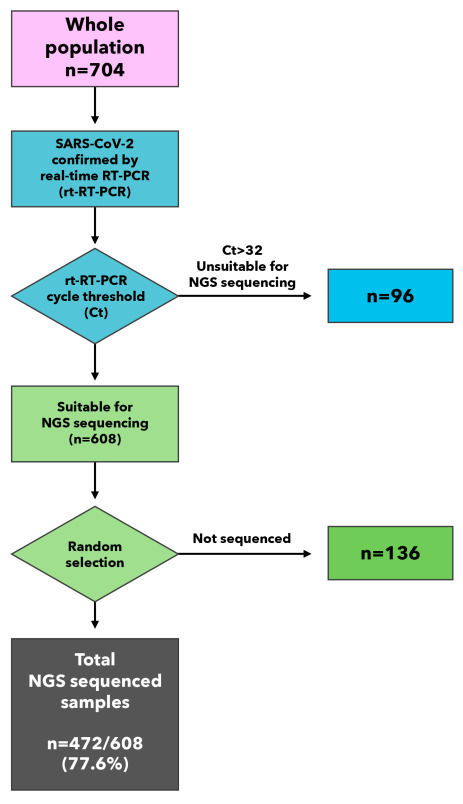
Flowchart describing the selection procedure for next-generation sequencing. rt-RT-PCR – real-time reverse transcription polymerase chain reaction.

**Figure 3:**
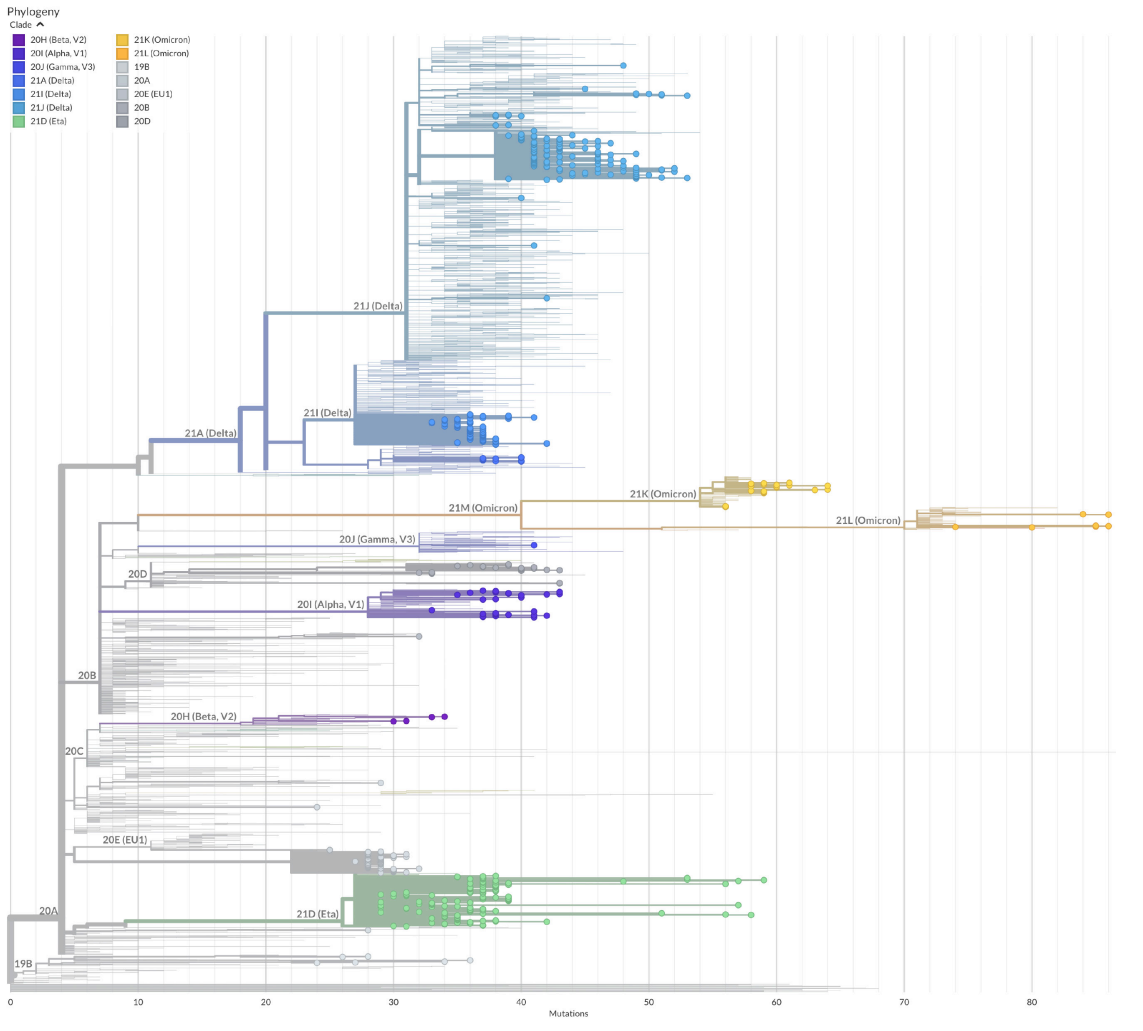
Nextclade phylogenetic tree of SARS-CoV2 genomes compared to reference sequences retrieved from GISAID, according to identified clades. Branches with coloured dots refer to sequences obtained from the convenience sample of migrants.

**Figure 4:**
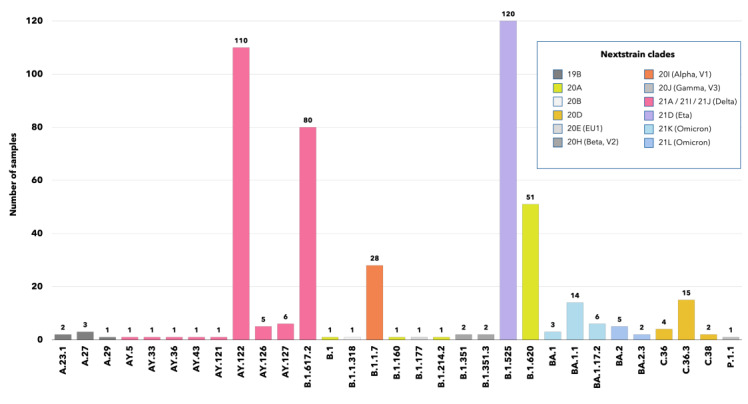
Description of the SARS-CoV2 clades and lineages identified in the convenience sample of migrants, according to Nextclade and the Pangolin COVID-19 tools. Values plotted on top of each bar indicate the number of SARS-CoV-2 strains belonging to each lineage.

Of the 608 specimens eligible for next-generation sequencing (**Figure 2**), we randomly selected 472 and obtained their whole-genome sequences. By using the Nextclade tool, we carried out a preliminary phylogenetic analysis to identify groups of genomes with a common evolutionary profile, as well as potentially divergent genomes (**Figure 3**). Altogether, we identified 12 unique clades belonging to 31 different lineages/sub-lineages (**Figure 4**). The sub-lineage AY.122 was present in more than half of SARS-CoV-2 delta variants (n/N = 110/206), followed by the original Delta lineage B.1.617.2 (n/N = 80/206) and several other sub-lineages (AY.5, AY.33, AY.36, AY.43, AY.121, AY.126 and AY.127) with a much lower prevalence. The clade 21D, corresponding to the lineage B.1.525 (eta), was isolated in 120 samples, while 54 viral genomes belonged to the clade 20A and were classified by Pangolin as B.1.620 (n = 51), B.1 (n = 1), B.1.160 (n = 1), and B.1.214.2 (n = 1) (**Figure 4**).

The omicron variant was present in 30 genomes with two clades, 21L (Pango lineages BA.1 and BA.1.1) and 21K (lineages BA.2 and BA.2.3), represented in 23 and 7 samples, respectively (**Figure 4**). Moreover, Nextclade assigned 28 genomes to the variant Alpha, V1 (clade 20I, lineage B.1.1.7), while 21 sequences were classified within the clade 20D belonging to lineages C.36.3 (n = 12), C.36 (n = 7) and C.38 (n = 2). Lastly, 16 genomes were assigned to clades 19B (A.23.1, n = 2; A.27, n = 3; A.29, n = 1), 20H (beta, V2) (B.1.351, n = 2; B.1.351.3, n = 2), 20J (gamma, V3) (P.1.1, n = 1), 20B (B.1.1.318, n = 1), and 20E (EU1) (B.1.177, n = 1).

**Figure 5:**
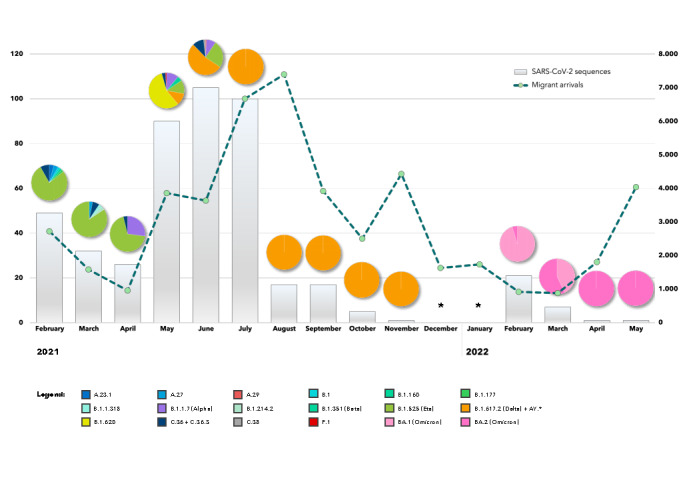
Monthly distribution of the SARS-CoV-2 lineages detected during the experimental molecular surveillance.

The distribution of the SARS-CoV-2 lineages over time (**Figure 5**) highlighted an evolving epidemic among migrants under monitoring: the lineage B.1.525 (eta) was prevalent in the first trimester of surveillance, while the alpha variant (lineage B.1.1.7) was detected between March and June 2021, but never reaching a predominant role. The B.1.620 lineage prevailed in May 2021 only, with delta lineages (B.1.617.2 + AY.*) being first detected in the same month, then becoming prevalent and unique until November 2021, until they were eventually substituted from February 2022 onwards by the emerging omicron variants (**Figure 5**).

**Table 2** shows the first detection dates of selected SARS-CoV-2 lineages found in migrants landing in Sicily and its smaller islands, and their circulation at the global, European, and Italian levels. A.23.1 was identified for the first time in June 2020 in the Democratic Republic of Congo and rapidly documented in neighbouring countries such as Uganda, Rwanda, and Kenya, after which it entered Europe in November 2020. Notably, A.23.1 was infrequently reported in Europe (n = 259). The first of the only two records from Italy found in GISAID dated back to the beginning of February 2021, immediately followed by two more A.23.1 virus strains identified in migrants originating from Egypt and landing in Sicily in the same month.

**Table 2:** Comparison at different geographic levels of first detection dates and place of detection (country/region) of selected SARS-CoV-2 lineages using GISAID database

	World*		Europe†		Italy		Italian borders‡	
**Lineage**	**Number of detections**	**First detection date and country/region**	**Number of detections**	**First detection date and country/region**	**Number of detections**	**First detection date and country/region**	**Number of detections**	**First detection date and country/region**
A.23.1	1347	8 June 2020, Democratic Republic of Congo	259	27 November 2020, UK	2	8 February 2021, Lombardy	2	24 February 2021, Egypt
A.27	832	26 November 2020, Senegal	491	14 December 2020, Denmark	10	17 February 2021, Sardinia	3	19 February 2021, Bangladesh
A.29	173	21 August 2020, Gambia	121	19 February 2021, Norway	-	-	1	15 June 2021, Syria
B.1.620	1370	5 April 2020, Senegal	606	5 February 2021, France	11	15 April 2021, Apulia	51	3 May 2021, Burkina Faso

*Excluding Europe.

†Excluding Italy.

‡Migrants tested at disembarkation and/or at reception facilities.

A.27 was initially observed in November 2020 in the Saharan countries, entering Northern Europe through Denmark, the Netherlands, and the UK in December 2020. The lineage was rarely reported in Italy, being first notified in Sardinia, the second major island in the Mediterranean Sea, and concurrently found among migrants (the first one coming from Bangladesh) who landed in Sicily in mid-February 2021.

As verified through GISAID, the lineage A.29 was primarily reported in Gambia in August 2020. Only 173 genomes with a complete collection date were released globally, of which nearly 70% were from Norway (first detection on 19 February 2021) and the UK This variant seemingly did not circulate in Italy at all before the unique genome became publicly available, identified through the SAMI-Surv system in a migrant subject originating from Syria in mid-June 2021.

Lastly, the lineage B.1.620 mostly originated from Senegal between April and May 2020 (first detection on 5 April 2020), after which it spread across the African continent from December 2020 onwards. In Europe, this lineage was first reported in France about one year later (5 February 2021), then propagating in several countries, including Italy, where it was first detected in Apulia in mid-April 2021 and immediately after reported in Sicily in early May 2021.

The mutation analysis allowed us to identify 786 unique coding mutations: 617 amino acid (AA) substitutions, 156 amino acid deletions, 7 stop codons, and 6 insertions.

**Figure 6:**
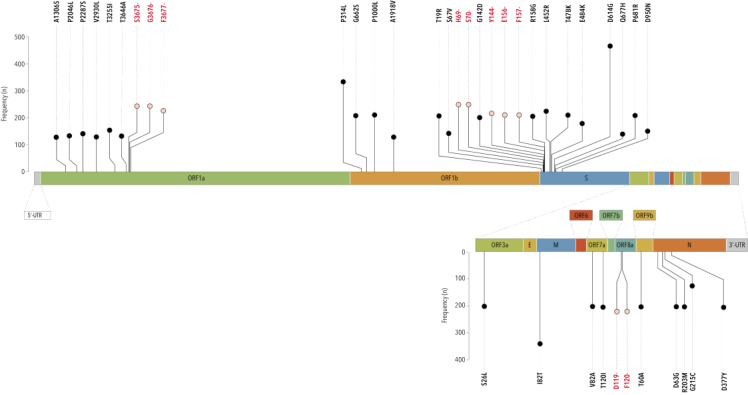
Genomic location and frequency distribution of top 40 non-synonymous mutations found in SARS-CoV-2 genomes sampled in the migrant subjects.

Overall, nine mutations were recurrently documented (≥45% of genomes), four were AA substitutions (S:D614G, 98.7%; M:I82T, 72.9%; ORF1b:P314L, 70.6% and S:L452R, 47.5%), three were adjacent AA deletions G3675-, G3676-, G3677- (50.0%) in the ORF1a gene, and two were adjacent AA deletions in the spike protein (S:H69- and S:H70-, 49.9%) (**Figure 6**; Table S2 in the **Online Supplementary document**).

**Figure 7:**
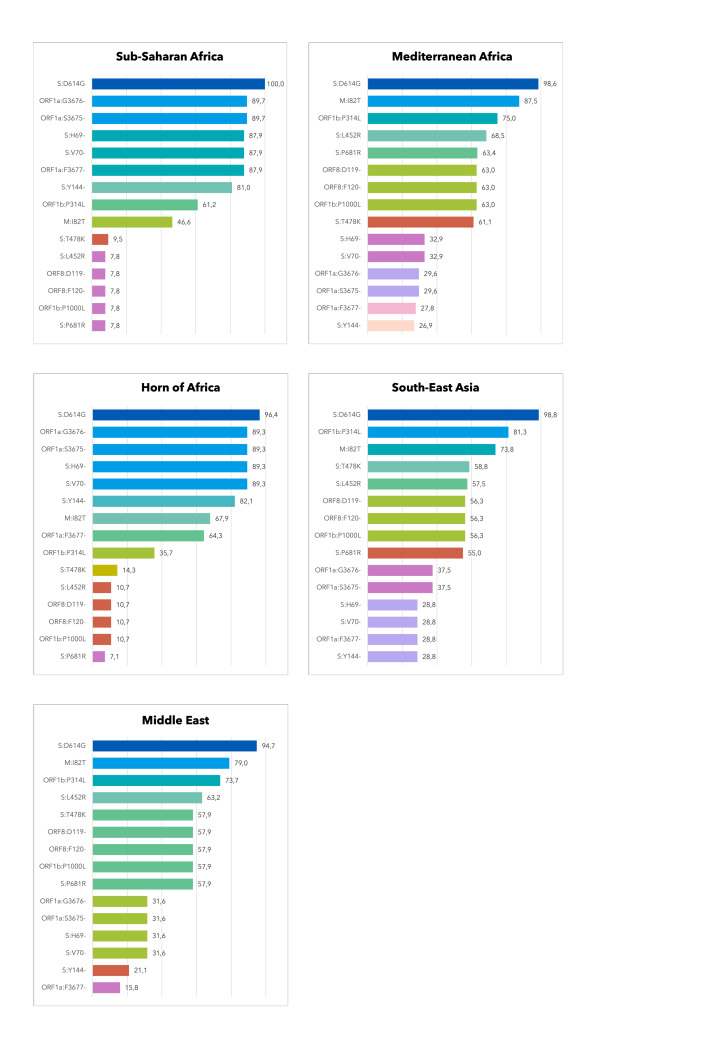
Distribution of the 15 most prevalent SARS-CoV-2 non-synonymous substitutions detected in the convenience sample of migrants by geographic macro-area of origin.

In general, S:D614G was widely found in all geographic areas (range: 94.7% - 100.0%) and very early fixed globally in the pandemic (**Figure 7**, **Table 3**). In the context of the spike protein, the double AA deletion S:H69- and S:V70- prevailed in the Horn of Africa (89.3%) and in sub-Saharan Africa (87.9%), while it was detected in a much lesser extent in the other regions (range: 28.8% to 32.9% of genomes). From the perspective of the time-period, viruses carrying these two AA deletions mostly circulated between February and June 2021 with the alpha variant (B.1.1.7), disappeared for about a semester, and thereafter reoccurred between February and March 2022 with the first omicron variant (BA.1). We observed a similar pattern for the AA deletion S:Y144-.

**Table 3:** Frequency distribution of top-15 non-synonymous mutations found in SARS-CoV-2 genomes sampled in the convenience sample of migrants, according to year and month of sampling

	2021										2022			
**Top 15**	**February**	**March**	**April**	**May**	**June**	**July**	**August**	**September**	**October**	**November**	**February**	**March**	**April**	**May**
S:D614G	91.84	96.88	100.00	100.00	100.00	100.00	100.00	100.00	100.00	100.00	100.00	100.00	100.00	100.00
S:H69-	73.47	90.62	100.00	83.33	43.81	0.00	0.00	0.00	0.00	0.00	85.71	42.86	0.00	0.00
S:V70-	73.47	90.62	100.00	83.33	43.81	0.00	0.00	0.00	0.00	0.00	85.71	42.86	0.00	0.00
S:L452R	4.08	3.12	3.85	14.44	63.81	100.00	100.00	100.00	100.00	100.00	100.00	100.00	100.00	100.00
S:T478K	0.00	0.00	0.00	11.11	51.43	98.00	94.12	10.00	100.00	100.00	38.10	0.00	100.00	0.00
S:P681R	4.08	0.00	0.00	11.11	53.33	100.00	100.00	100.00	100.00	100.00	100.00	100.00	100.00	100.00
S:Y144-	77.55	87.50	88.46	75.56	31.43	0.00	0.00	0.00	0.00	0.00	71.43	28.57	0.00	0.00
M:I82T	77.55	87.50	73.08	26.67	90.48	100.00	100.00	100.00	100.00	100.00	100.00	100.00	100.00	100.00
ORF1a:S3675-	77.55	96.88	96.15	85.56	33.33	0.00	0.00	0.00	0.00	0.00	100.00	100.00	100.00	100.00
ORF1a:G3676-	77.55	96.88	96.15	85.56	33.33	0.00	0.00	0.00	0.00	0.00	100.00	100.00	100.00	100.00
ORF1a:F3677-	77.55	96.88	96.15	85.56	33.33	0.00	0.00	0.00	0.00	0.00	4.76	57.14	100.00	100.00
ORF1b:P314L	12.24	12.50	26.92	87.78	70.48	95.00	100.00	88.24	100.00	100.00	100.00	100.00	100.00	100.00
ORF1b:P1000L	4.08	3.12	0.00	11.11	53.33	100.00	100.00	100.00	100.00	100.00	0.00	0.00	0.00	0.00
ORF8:D119-	4.08	3.12	0.00	11.11	53.33	100.00	100.00	100.00	100.00	100.00	0.00	0.00	0.00	0.00
ORF8:F120-	4.08	3.12	0.00	11.11	53.33	100.00	100.00	100.00	100.00	100.00	0.00	0.00	0.00	0.00

Meanwhile, the S:T478K and S:L452R mutations were documented in nearly 60% of viral strains from South-East Asia and the Middle East, as also observed for S:L452R and S:P681R in Mediterranean Africa. The spread of these mutations increased significantly from June 2021 onwards, thus being fixed in almost all SARS-CoV-2 strains. The mutations I82T (in the M gene) and P314L (in the ORF1a gene) were found in about 80% of genomes from the geographic belt, including the Mediterranean region of Africa, the Middle East, and Southeast Asian countries, where M:I82T was rapidly stable and ORF1b:P314L became detectable in almost all genomes from July 2021 onwards.

Lastly, the three adjacent AA deletions in ORF1a (S3675-, G3676-, and F3677-) showed the largest spread, especially in sub-Saharan Africa and the Horn of Africa. They seem to have co-evolved following a trend similar to that of the S:H69- and S:V70- over 2021, reappearing in 2022 and then recurring in every genome.

## DISCUSSION

The role of genomic surveillance in generating relevant data for tracking pathogen transmission and evolution, as well as providing real-time data for national and international public health response became exceedingly clear from the first phase of the pandemic [25]. Consequently, international efforts towards open data sharing were promoted, enabling almost real-time monitoring of the evolution of SARS-CoV-2 globally based on shared knowledge that informed public health interventions.

To date, whole genome sequencing remains the gold standard for variant surveillance, as it can unambiguously identify known variants and lineages or detect new mutations/escape mutants as they arise. The World Health Organization (WHO) has highlighted an urgent need for the global adoption of whole genome sequencing, aiming to fill the gap in sequencing availability (especially in low-income countries) and to improve countries’ public health decision-making abilities that are key to managing the ongoing pandemic and preventing future outbreaks.

In this context, a coordinated network of WGS laboratories named ‘I.Co.Gen.’ (Italian-COVID-19-Genomic) was implemented in Italy on 29 April 2021 to monitor both the SARS-CoV-2 variants and other pathogens relevant to public health.

The SARS-CoV-2 molecular epidemiologic surveillance programme for migrants, refugees, and asylum seekers arriving to Europe via Italy after crossing the Mediterranean Sea offered an important insight into the molecular characteristics and the genetic diversity of SARS-CoV-2 genomes, providing evidence in support of dedicated public health interventions.

Per the official statistics on migration movements provided by the Italian government [10], our sample was representative of a predominantly male population. While men, mainly in the working age, usually leave their country to find a job and a better future, women and their children move later to join their husbands.

In our sample, almost all the individuals testing positive at the landing were pauci-symptomatic or asymptomatic, a clinical status that could be partially explained by the sample’s low median age. This affirms the importance of massive SARS-CoV-2 screening at disembarkment and supports the implementation of dedicated quarantine areas to temporarily host migrants before they can share common spaces with prior arrivals and the personnel overseeing them within reception centres. However, more structured evidence is needed to determine if quarantining may effectively limit the transmission of SARS-CoV-2 in such settings or may bring any additional protection for the general population beyond the levels that could be achieved by conventional containment and protection measures [26].

Second, in line with the official statistics [10], the migrants’ countries of origin mainly in the Mediterranean basin and sub-Saharan Africa, followed by Southeast Asia, especially Bangladesh. Therefore, we assumed that the molecular epidemiological approach proposed in our experience may add knowledge on transmission dynamics in relation to migratory paths and flows and, to some extent, provide information on the circulation of SARS-CoV-2 variants in the countries of origin.

Based on the overview of the distribution of the SARS-CoV-2 lineages detected over time, the experimental surveillance system presented here captured an evolving molecular scenario. In the first surveillance period, there was a dynamic evolution and a heterogeneous circulation of the pandemic viral lineages, with several new variants of interest and VOCs concentrated in a short time. Looking at the variability of SARS-CoV-2, the genomes obtained belonged to 12 unique clades and 31 different lineages which varied over time; most followed the trends observed at the European level and globally. Differently, some lineages like B.1.525 (eta), a variant designated under investigation (a variant of interest) up to 3 September 2021 [27] and sharing several relevant mutations in the spike protein ž with other VOCs, prevailed among migrant populations in a given period, such as the first semester of 2021, when the variant Alpha (B.1.1.7) became dominant in Italy [28] and elsewhere [29]. Notably, a wide spread of B.1.525 with very low level of B.1.1.7 was reported in Libya in early 2021 [30], as documented in other West African countries [31].

Nevertheless, some of the lineages identified among migrants arriving in Sicily and its smaller islands circulated at extremely low levels and, in some cases, were aligned to or precedent to their introduction into Italy, as was the case for A.23.1, A.27, A.29, and B.1.620. Generally initially identified in countries of sub-Saharan Africa, a few hundred genomes belonging to these lineages were documented worldwide. The sub-lineage A.23.1, first reported in two prisoners, predominated in Uganda between December 2020 and January 2021 [32]. It was characterised by the three spike mutations F157L, V367F, and Q613H by acquiring additional AA substitutions in ORF1a, ORF8, and ORF9, as well as P681R in the spike protein. This latter mutation also occurred in the globally dominant delta variant (B.1.617.2) and relative AY sub-lineages which emerged several months later, suggesting a greater advantage in the transmissibility for viruses encoding this substitution [33,34]. This variant quickly spread in the general population, dominating the local epidemic, and was then exported into neighbouring countries such as Rwanda and Kenya [35]. A phylogeographic reconstruction suggested two different patterns of the introduction of this sub-lineage into other African countries: the first one into South Africa, which likely occurred directly through a subject who had travelled to Kenya, and the second one which was likely introduced into Ghana back via Europe [31]. This lineage was seldom encountered in Italy [36] and has no longer been detected across it since August 2021.

The genomes belonging to the lineage A.27 hold a combination of key mutations, either AA deletions or substitutions [37], especially in the spike protein, which bring significant concerns due to the documented ability to increase viral transmission or to escape immune response, with potential impact on vaccine-based preventive measures [38]. Among these, N501Y, L452R, and H655Y also carried by other variants which have dominated the most recent periods of the pandemic, including the omicron BA.5, suggesting a consistent advantage in terms of viral fitness and transmissibility [39]. Only a few units have been identified in Italy, with other researchers observing this lineage in a family cluster of autochthonous inhabitants of Sardinia, the second-largest island in the Mediterranean Sea [40], in the same period as we observed here. Even though no data was available on the possible contacts, differences were found in the panel of nucleotide substitutions, supporting the hypothesis that two distinct patterns of introduction of this lineage occurred in our country.

Regarding the lineage A.29, barely 173 genomes have been submitted to GISAID. Originating in Gambia, an important transit country for irregular migrants from sub-Saharan Africa *en route* to Europe, this lineage circulated for just one year (from August 2020 to August 2021), mostly in Northern Europe. Compared to A.27, the A.29 lineage showed distinct mutations in spike, some of which (Y449H, N501Y, and H665Y) were shared by VOCs. Interestingly, the single A.29 genome identified in a migrant originating from Syria landing in Sicily and not previously documented in Italy did not show the peculiar deletions L141- and Y144-, as similarly observed in a homogeneous cluster of sequences collected in neighbouring Turkey in August 2021, right before the lineage became no longer detectable globally. Unfortunately, existing reports cannot explain the potential evolutionary advantage of this genomic profile to the virus.

Lastly, the B.1.620 lineage was first detected in Europe in early February 2021 and gained attention because it bore multiple mutations and deletions (mostly in the spike protein) in common with widespread VOCs, such as S477N, E484K, P681H, HV69/70-, Y144-, and LLA241/243-, together with a set of uncharacterised AA substitutions in other genes. Responsible for a large cluster of cases in Europe [41], the presumed origin of this lineage was Central Africa; it was then introduced into other geographic areas on multiple occasions, as highlighted through our experimental molecular surveillance system. Overall, the genomes analysed showed many unique coding mutations, either in terms of AA substitutions or AA deletions. As a response to evolutionary pressure, viral genomes dynamically evolved over time. According to our findings, the total number of mutations per genome increased throughout the study period, sharing a trend towards higher mutational load among emergent viral strains, as noted with the predominance of delta and omicron variants [34,39].

Some mutations were particularly common in our sample. This is the case for D614G in the spike protein, which was detected in 98.7% of SARS-CoV-2 sequences, irrespective of the viral lineage. This specific mutation rapidly fixed at the population level worldwide, suggesting a role in viral entry through the enhancement of interaction between the receptor-binding-domain of the S protein and the entry receptor ACE2, offering to the virus a selective advantage that made it globally dominant. Other mutations frequently occurred, some of which have been associated with reduced neutralisation by antibodies from acquired immunity, like L452R [41], or which were able to affect RdRp activity and thus viral RNA replication and infectivity, such as ORF1b:P314L [42].

Furthermore, the lineage B.1.525, which was largely identified among migrants, carried some AA deletions such as ORF1a:SGF3675/3677- or S:HV69/70-, clearly anticipating the occurrence in VOCs which dominated the pandemic in the early phase, like alpha (B.1.1.7) or the more recent omicron (BA.*).

Ultimately, besides the temporal dynamics of such mutations, our findings showed geographical patterns in the distribution of some mutational profiles, specifically occurring in the countries of origin of the migrants under monitoring because of the high circulation of some strains in those areas.

Our results should be interpreted in light of some limitations, including the convenience sample that has not been continuously available over the whole period examined and the different epidemic waves characterised by several predominant variants. Specifically, we cannot exclude that among the samples unsuitable for sequencing or not included because of the convenience sampling strategy adopted, there might have been some viral variants or mutational patterns of potential public health interest. Furthermore, the incompleteness of some self-reported data, i.e. on migration routes and clinical or vaccination status, will hopefully be overcome in the future through the implementation of a dedicated digital platform interoperable with the border surveillance system. This will also allow us to obtain reliable estimates on SARS-CoV-2 incidence in migrants arriving in Sicily and hosted in dedicated facilities.

Further, we carried out this study in an emergency operative setting, which presented various challenges. Primarily, logistical difficulties arose due to the limited laboratory capacities on the small island of Lampedusa, where most of the migrants in our study arrived. Consequently, samples had to be transported by air. Additionally, tracking the migrants until the completion of their isolation proved challenging as they were relocated to different centres. Furthermore, due to the emergency circumstances, we could not always obtain a representative sample.

Despite the aforementioned limitations, this study is, to the best of our knowledge, the first structured attempt across the pandemic to screen irregular migrants upon arrival in transit/destination countries, as no comparable evidence is available on these vulnerable groups to date. As the risk of infection among migrants, refugees, and asylum seekers is significantly higher than in the general population, this often generates grievances among these enclosed groups and tensions with host communities [43].

The management of COVID-19 risk for newly arrived migrants hosted in reception centres presents numerous challenges, mainly related to limited spaces, cultural and linguistic barriers, and heterogeneity in COVID-19 risk perception and compliance with preventive measures for reducing the risk of transmission [44]. Moreover, refugees, migrants, and asylum seekers have structural vulnerabilities, including fear of contacting the health care system, cultural differences, discrimination, health illiteracy, and a lack of readily available and culturally appropriate educational materials [45]. This leaves a need to include these vulnerable groups in the national response plans for reducing SARS-CoV-2 transmission and for the COVID-19 vaccine rollout. To this end, some high-income countries in the WHO European region, including Italy, have already implemented specific programmes to extend COVID-19 vaccination to irregular migrants and some other countries in the WHO African and Eastern Mediterranean regions are experiencing the same challenges [46].

Lastly, migration has been mentioned as a factor contributing to or resulting in new ecological niches for emerging and re-emerging infectious diseases [47]. Establishing a genome-based surveillance system dedicated to migration routes from areas of the globe characterised by the circulation of highly transmissible or potentially pandemic microorganisms may support preparedness response, thereby strengthening public health measures. Simultaneously, this may add knowledge on the evolutionary patterns of pathogens endemic in areas with limited access to genome sequencing facilities, especially in the case of emerging variants or escape mutants with possible impact on the transmissibility, severity, and immunity. Lastly, this should also include factors related to climate change that have been associated with the appearance and resurgence of vector-borne infectious diseases [48].

## CONCLUSIONS

The implementation of genomic-based surveillance on emerging SARS-CoV-2 variants in low-income countries with inadequate testing and sequencing capabilities, as well as limited vaccine storage capacities, may help inform public health interventions at a global level [7]. However, there is little evidence to date on molecular investigations of SARS-CoV-2 variants from areas where they might emerge [49,50]. While confirming our preliminary findings [51], we provided further evidence on the value of molecular surveillance ofs migration movements to Europe from low-resource countries to improve the knowledge of the global SARS-CoV-2 transmission dynamics. This approach will allow us to forecast future epidemiological scenarios related to the emergence of unknown variants and their potential impact on the effectiveness of currently available vaccines that need to be re-engineered over time [14]. It may also provide additional data on potential genetic signatures and evolutionary trajectories of certain lineages showing altered antigenic properties, increased transmissibility, and higher disease severity [52].

Moreover, a continuous comparison between the detection dates of SARS-CoV-2 lineages isolated in migrants crossing the Mediterranean Sea and their circulation at the national, European, and global scales may offer insights into the introduction of new variants through migration routes and on cases imported by individuals visiting low-resources areas. This evidence may also be of interest for the upcoming implementation of the WHO Global Genomic Surveillance Strategy for Pathogens with Pandemic and Epidemic Potential [53].

Due to the above-mentioned reasons, the proposed genomic-based experimental surveillance model might be integrated with the ones already in place for the early detection of COVID-19 cases at points of entry in Europe, including dedicated reception facilities and ground crossings [13]. It could be extended to other emergent or re-emergent pathogens [3] or exported to areas with high immigration flows from lower-resource countries.

Based on our research experience, creating a molecular surveillance programme specifically for newly arriving migrants during an epidemic or pre-pandemic can have a crucial impact on protecting public health, advancing health equity, and strengthening preparedness and response efforts across borders. Several public health policy implications can be emphasised from different viewpoints:

− Early detection and response: early detection of pathogens can prompt rapid public health responses such as targeted testing, contact tracing, and quarantine measures to prevent potential outbreaks within migrant communities and mitigate further transmission to the host population;
− Tailored healthcare services: understanding the prevalence and dynamics of potential pandemic pathogens among migrant populations can inform the development of tailored healthcare services, including access to testing, treatment, and vaccination services, which are crucial for their health and well-being and for controlling the spread within migrant communities and beyond;
− Equitable distribution of resources: molecular surveillance data can guide the equitable distribution of resources (e.g. testing kits, personal protective equipment) and healthcare personnel to points of entry and host centres, ensuring that migrant populations receive adequate support and enough resources to prevent and manage pathogens transmission effectively;
− Cross-border collaboration: sharing surveillance data and best practices can facilitate coordinated responses across different regions and countries with a route-based approach, enhancing overall public health outcomes. To this end, a cross-border collaboration, including information sharing among European countries and with countries of transit and origin, is essential;
− Access to health and addressing health inequalities: migrants, refugees, and asylum seekers often face structural barriers to healthcare access, including language barriers, legal restrictions, and socio-economic inequalities. The operational implementation of surveillance can help identify and address these barriers, while detecting any other health needs at an early stage so they can promptly be addressed, therefore ensuring equitable access to healthcare services and fostering health equity among migrant populations.

Our findings suggest the need for a coordinated, international public health effort to provide specific COVID-19 vaccination programmes for irregular migrants besides the ones already in place, to ensure equitable access to vaccines in low-income countries having inadequate testing and sequencing capabilities [54].

## Additional material


Online Supplementary Document
