# Supplementary material for: SARS-CoV-2 genomic surveillance of migrants arriving to Europe through the Mediterranean routes
Source: J Glob Health. 2024 Jul 5;14:05017. doi: 10.7189/jogh.14.05017 (PMC11223754; doi:10.7189/jogh.14.05017)
Supplement: Online Supplementary Document [file jogh-14-05017-s001.pdf]

**Table S1.** Migrant's countries of origin

| Country | Macro Area | n=672 |
|---|---|---|
| Afghanistan | Middle East | 7 |
| Algeria | Mediterranean Africa | 4 |
| Bangladesh | South-East Asia | 127 |
| Burkina Faso | Sub-Saharan Africa | 14 |
| Cameroon | Sub-Saharan Africa | 12 |
| Ivory coast | Sub-Saharan Africa | 33 |
| Egypt | Mediterranean Africa | 86 |
| Eritrea | Horn of Africa | 20 |
| Ethiopia | Horn of Africa | 8 |
| Gambia | Sub-Saharan Africa | 7 |
| Ghana | Sub-Saharan Africa | 9 |
| Guinea | Sub-Saharan Africa | 3 |
| Guinea Conakry | Sub-Saharan Africa | 19 |
| Indonesia | South-East Asia | 1 |
| Iran | Middle East | 13 |
| Kenya | Horn of Africa | 1 |
| Libya | Mediterranean Africa | 1 |
| Mali | Sub-Saharan Africa | 14 |
| Morocco | Mediterranean Africa | 30 |
| Niger | Sub-Saharan Africa | 1 |
| Nigeria | Sub-Saharan Africa | 6 |
| Pakistan | Middle East | 5 |
| Senegal | Sub-Saharan Africa | 5 |
| Sierra Leone | Sub-Saharan Africa | 3 |
| Syria | Middle East | 19 |
| Somalia | Horn of Africa | 11 |

| | | |
|---|---|---|
| Sudan | Sub-Saharan Africa | 22 |
| Togo | Sub-Saharan Africa | 2 |
| Tunisia | Mediterranean Africa | 188 |
| Yemen | Middle East | 1 |

**Table S2.** GISAID results

| Sequence name | GISAID accession number | Collection date | Age (years) | Sex | Country of origin | Pangolin_lineage |
|---|---|---|---|---|---|---|
| hCoV-19/Italy/SIC-AOUP-UNIPA_59323/2021 | EPI_ISL_13620679 | 23/2/2021 | 19 | M | Sudan | B.1 |
| hCoV-19/Italy/SIC-AOUP-UNIPA_59324/2021 | EPI_ISL_13637845 | 23/2/2021 | 27 | M | Egypt | B.1.525 |
| hCoV-19/Italy/SIC-AOUP-UNIPA_59325/2021 | EPI_ISL_13637846 | 23/2/2021 | 22 | M | Morocco | B.1.525 |
| hCoV-19/Italy/SIC-AOUP-UNIPA_59328/2021 | EPI_ISL_13637847 | 23/2/2021 | 18 | M | Egypt | B.1.525 |
| hCoV-19/Italy/SIC-AOUP-UNIPA_59329/2021 | EPI_ISL_13637848 | 23/2/2021 | 35 | M | Morocco | B.1.525 |
| hCoV-19/Italy/SIC-AOUP-UNIPA_59330/2021 | EPI_ISL_13637849 | 23/2/2021 | 23 | M | Egypt | B.1.525 |
| hCoV-19/Italy/SIC-AOUP-UNIPA_59331/2021 | EPI_ISL_13637850 | 23/2/2021 | 34 | M | Egypt | B.1.525 |
| hCoV-19/Italy/SIC-AOUP-UNIPA_59332/2021 | EPI_ISL_13637851 | 23/2/2021 | 26 | M | Egypt | B.1.525 |
| hCoV-19/Italy/SIC-AOUP-UNIPA_59333/2021 | EPI_ISL_13637852 | 23/2/2021 | 25 | M | Egypt | B.1.525 |
| hCoV-19/Italy/SIC-AOUP-UNIPA_59334/2021 | EPI_ISL_13637853 | 23/2/2021 | 25 | M | Egypt | B.1.525 |
| hCoV-19/Italy/SIC-AOUP-UNIPA_59335/2021 | EPI_ISL_13637854 | 23/2/2021 | 18 | M | Somalia | B.1.525 |
| hCoV-19/Italy/SIC-AOUP-UNIPA_59336/2021 | EPI_ISL_13637855 | 23/2/2021 | 18 | M | Somalia | B.1.525 |
| hCoV-19/Italy/SIC-AOUP-UNIPA_59337/2021 | EPI_ISL_13637856 | 23/2/2021 | 44 | M | Somalia | B.1.525 |
| hCoV-19/Italy/SIC-AOUP-UNIPA_59338/2021 | EPI_ISL_13637857 | 23/2/2021 | 23 | M | Somalia | B.1.525 |
| hCoV-19/Italy/SIC-AOUP-UNIPA_59339/2021 | EPI_ISL_13637858 | 23/2/2021 | 16 | M | Egypt | B.1.525 |
| hCoV-19/Italy/SIC-AOUP-UNIPA_59340/2021 | EPI_ISL_13637859 | 23/2/2021 | 27 | M | Egypt | B.1.525 |
| hCoV-19/Italy/SIC-AOUP-UNIPA_59341/2021 | EPI_ISL_13637860 | 23/2/2021 | 37 | M | Egypt | B.1.525 |
| hCoV-19/Italy/SIC-AOUP-UNIPA_59342/2021 | EPI_ISL_1585281 | 23/2/2021 | 26 | M | Egypt | A.23.1 |
| hCoV-19/Italy/SIC-AOUP-UNIPA_59344/2021 | EPI_ISL_13637861 | 23/2/2021 | 28 | M | Egypt | B.1.525 |
| hCoV-19/Italy/SIC-AOUP-UNIPA_59345/2021 | EPI_ISL_13637862 | 23/2/2021 | 26 | M | Guinea Conakry | B.1.525 |

| | | | | | | |
|---|---|---|---|---|---|---|
| hCoV-19/Italy/SIC-AOUP-UNIPA_59346/2021 | EPI_ISL_13637863 | 23/2/2021 | 18 | M | Somalia | B.1.525 |
| hCoV-19/Italy/SIC-AOUP-UNIPA_59347/2021 | EPI_ISL_13637864 | 23/2/2021 | 18 | M | Somalia | B.1.525 |
| hCoV-19/Italy/SIC-AOUP-UNIPA_59348/2021 | EPI_ISL_1585282 | 23/2/2021 | 19 | M | Egypt | A.23.1 |
| hCoV-19/Italy/SIC-AOUP-UNIPA_59501/2021 | EPI_ISL_13637865 | 19/2/2021 | 17 | M | Bangladesh | C.36 |
| hCoV-19/Italy/SIC-AOUP-UNIPA_59503/2021 | EPI_ISL_13637866 | 19/2/2021 | 30 | M | Bangladesh | B.1.525 |
| hCoV-19/Italy/SIC-AOUP-UNIPA_59504/2021 | EPI_ISL_13637867 | 19/2/2021 | 29 | M | Bangladesh | C.36 |
| hCoV-19/Italy/SIC-AOUP-UNIPA_59505/2021 | EPI_ISL_13637868 | 19/2/2021 | 29 | M | Egypt | B.1.525 |
| hCoV-19/Italy/SIC-AOUP-UNIPA_59506/2021 | EPI_ISL_13637869 | 19/2/2021 | 48 | F | Morocco | B.1.525 |
| hCoV-19/Italy/SIC-AOUP-UNIPA_59507/2021 | EPI_ISL_13637870 | 19/2/2021 | 29 | M | Bangladesh | B.1.525 |
| hCoV-19/Italy/SIC-AOUP-UNIPA_59508/2021 | EPI_ISL_13637871 | 19/2/2021 | 34 | M | Bangladesh | B.1.525 |
| hCoV-19/Italy/SIC-AOUP-UNIPA_59509/2021 | EPI_ISL_13637872 | 19/2/2021 | 0 | M | Bangladesh | C.36 |
| hCoV-19/Italy/SIC-AOUP-UNIPA_59510/2021 | EPI_ISL_13637873 | 19/2/2021 | 24 | M | Bangladesh | B.1.525 |
| hCoV-19/Italy/SIC-AOUP-UNIPA_59511/2021 | EPI_ISL_13637874 | 19/2/2021 | 28 | M | Bangladesh | B.1.525 |
| hCoV-19/Italy/SIC-AOUP-UNIPA_59512/2021 | EPI_ISL_13637875 | 19/2/2021 | 19 | M | Bangladesh | C.36 |
| hCoV-19/Italy/SIC-AOUP-UNIPA_59513/2021 | EPI_ISL_13637876 | 19/2/2021 | 34 | M | Bangladesh | B.1.525 |
| hCoV-19/Italy/SIC-AOUP-UNIPA_59514/2021 | EPI_ISL_13637877 | 19/2/2021 | 24 | M | Morocco | B.1.525 |
| hCoV-19/Italy/SIC-AOUP-UNIPA_59515/2021 | EPI_ISL_13637878 | 19/2/2021 | 21 | M | Morocco | B.1.525 |
| hCoV-19/Italy/SIC-AOUP-UNIPA_59516/2021 | EPI_ISL_13637879 | 19/2/2021 | 25 | M | Morocco | B.1.525 |
| hCoV-19/Italy/SIC-AOUP-UNIPA_59517/2021 | EPI_ISL_13620676 | 18/2/2021 | 21 | M | Sierra Leone | B.1.177 |
| hCoV-19/Italy/SIC-AOUP-UNIPA_59518/2021 | EPI_ISL_13620677 | 18/2/2021 | 10 | F | Sierra Leone | B.1.160 |
| hCoV-19/Italy/SIC-AOUP-UNIPA_59519/2021 | EPI_ISL_13637880 | 19/2/2021 | 29 | M | Bangladesh | B.1.525 |

| | | | | | | |
|---|---|---|---|---|---|---|
| hCoV-19/Italy/SIC-AOUP-UNIPA_59520/2021 | EPI_ISL_13637881 | 19/2/2021 | 20 | M | Morocco | B.1.525 |
| hCoV-19/Italy/SIC-AOUP-UNIPA_59521/2021 | EPI_ISL_13637882 | 19/2/2021 | 37 | M | Bangladesh | B.1.525 |
| hCoV-19/Italy/SIC-AOUP-UNIPA_59522/2021 | EPI_ISL_13637883 | 19/2/2021 | 20 | M | Sudan | B.1.525 |
| hCoV-19/Italy/SIC-AOUP-UNIPA_59523/2021 | EPI_ISL_13637884 | 19/2/2021 | 21 | M | Mali | B.1.525 |
| hCoV-19/Italy/SIC-AOUP-UNIPA_59524/2021 | EPI_ISL_13637885 | 19/2/2021 | 36 | M | Pakistan | B.1.525 |
| hCoV-19/Italy/SIC-AOUP-UNIPA_59728/2021 | EPI_ISL_1315620 | 2/3/2021 | 31 | M | Ghana | B.1.525 |
| hCoV-19/Italy/SIC-AOUP-UNIPA_59743/2021 | EPI_ISL_13637886 | 27/2/2021 | 40 | M | Tunisia | B.1.525 |
| hCoV-19/Italy/SIC-AOUP-UNIPA_60566/2021 | EPI_ISL_1241860 | 7/3/2021 | 17 | M | Eritrea | B.1.525 |
| hCoV-19/Italy/SIC-AOUP-UNIPA_60567/2021 | EPI_ISL_13637887 | 7/3/2021 | 26 | M | Morocco | B.1.525 |
| hCoV-19/Italy/SIC-AOUP-UNIPA_60993/2021 | EPI_ISL_6202127 | 5/3/2021 | 39 | M | Ethiopia | B.1.1.7 |
| hCoV-19/Italy/SIC-AOUP-UNIPA_61022/2021 | EPI_ISL_6202115 | 8/3/2021 | 17 | F | Unknown | B.1.1.7 |
| hCoV-19/Italy/SIC-AOUP-UNIPA_61238/2021 | EPI_ISL_13637888 | 10/3/2021 | 37 | M | Tunisia | B.1.525 |
| hCoV-19/Italy/SIC-AOUP-UNIPA_61739/2021 | EPI_ISL_13531871 | 11/3/2021 | 21 | M | Eritrea | A.27 |
| hCoV-19/Italy/SIC-AOUP-UNIPA_61740/2021 | EPI_ISL_13637889 | 11/3/2021 | 21 | M | Eritrea | B.1.525 |
| hCoV-19/Italy/SIC-AOUP-UNIPA_61741/2021 | EPI_ISL_13637890 | 11/3/2021 | 44 | M | Syria | B.1.525 |
| hCoV-19/Italy/SIC-AOUP-UNIPA_61742/2021 | EPI_ISL_13637891 | 11/3/2021 | 26 | M | Egypt | B.1.525 |
| hCoV-19/Italy/SIC-AOUP-UNIPA_62534/2021 | EPI_ISL_13637892 | 12/3/2021 | 19 | M | Eritrea | B.1.525 |
| hCoV-19/Italy/SIC-AOUP-UNIPA_62535/2021 | EPI_ISL_13637893 | 12/3/2021 | 37 | M | Eritrea | B.1.525 |
| hCoV-19/Italy/SIC-AOUP-UNIPA_62851/2021 | EPI_ISL_13637894 | 16/3/2021 | 17 | M | Cameroon | B.1.525 |
| hCoV-19/Italy/SIC-AOUP-UNIPA_62854/2021 | EPI_ISL_13637895 | 16/3/2021 | 15 | M | Ivory Coast | B.1.525 |
| hCoV-19/Italy/SIC-AOUP-UNIPA_63313/2021 | EPI_ISL_13637896 | 21/3/2021 | 19 | M | Guinea Conakry | B.1.525 |
| hCoV-19/Italy/SIC-AOUP-UNIPA_64301/2021 | EPI_ISL_13620700 | 26/3/2021 | 18 | M | Ivory Coast | B.1.1.318 |

| | | | | | | |
|---|---|---|---|---|---|---|
| hCoV-19/Italy/SIC-AOUP-UNIPA_65645/2021 | EPI_ISL_13620682 | 26/3/2021 | 16 | M | Tunisia | B.1.214.2 |
| hCoV-19/Italy/SIC-AOUP-UNIPA_66141/2021 | EPI_ISL_13637897 | 8/4/2021 | 34 | F | Nigeria | B.1.525 |
| hCoV-19/Italy/SIC-AOUP-UNIPA_66142/2021 | EPI_ISL_13637898 | 26/3/2021 | 39 | M | Bangladesh | B.1.525 |
| hCoV-19/Italy/SIC-AOUP-UNIPA_66146/2021 | EPI_ISL_13637899 | 26/3/2021 | 24 | M | Egypt | B.1.525 |
| hCoV-19/Italy/SIC-AOUP-UNIPA_66147/2021 | EPI_ISL_13637900 | 2/4/2021 | 34 | M | Egypt | B.1.525 |
| hCoV-19/Italy/SIC-AOUP-UNIPA_66151/2021 | EPI_ISL_13637901 | 26/3/2021 | 38 | M | Ivory Coast | B.1.525 |
| hCoV-19/Italy/SIC-AOUP-UNIPA_66155/2021 | EPI_ISL_13637902 | 26/3/2021 | 18 | M | Bangladesh | B.1.525 |
| hCoV-19/Italy/SIC-AOUP-UNIPA_66156/2021 | EPI_ISL_13637903 | 18/3/2021 | 23 | M | Cameroon | B.1.525 |
| hCoV-19/Italy/SIC-AOUP-UNIPA_66157/2021 | EPI_ISL_13637904 | 17/3/2021 | 22 | M | Sudan | B.1.525 |
| hCoV-19/Italy/SIC-AOUP-UNIPA_66159/2021 | EPI_ISL_6202094 | 2/4/2021 | 21 | F | Guinea Conakry | B.1.525 |
| hCoV-19/Italy/SIC-AOUP-UNIPA_66161/2021 | EPI_ISL_13637905 | 17/3/2021 | 29 | M | Bangladesh | B.1.525 |
| hCoV-19/Italy/SIC-AOUP-UNIPA_66164/2021 | EPI_ISL_13637906 | 26/3/2021 | 20 | M | Tunisia | B.1.525 |
| hCoV-19/Italy/SIC-AOUP-UNIPA_66167/2021 | EPI_ISL_6202077 | 18/3/2021 | 29 | M | Morocco | B.1.525 |
| hCoV-19/Italy/SIC-AOUP-UNIPA_66185/2021 | EPI_ISL_13637907 | 31/3/2021 | 26 | M | Syria | B.1.525 |
| hCoV-19/Italy/SIC-AOUP-UNIPA_66186/2021 | EPI_ISL_13637908 | 31/3/2021 | 17 | F | Ivory Coast | B.1.525 |
| hCoV-19/Italy/SIC-AOUP-UNIPA_66187/2021 | EPI_ISL_13637909 | 31/3/2021 | 16 | M | Ghana | B.1.525 |
| hCoV-19/Italy/SIC-AOUP-UNIPA_66188/2021 | EPI_ISL_13637910 | 31/3/2021 | 33 | F | Cameroon | B.1.525 |
| hCoV-19/Italy/SIC-AOUP-UNIPA_66189/2021 | EPI_ISL_13637911 | 31/3/2021 | 17 | M | Ghana | B.1.525 |
| hCoV-19/Italy/SIC-AOUP-UNIPA_66190/2021 | EPI_ISL_13637912 | 31/3/2021 | 19 | M | Mali | B.1.525 |
| hCoV-19/Italy/SIC-AOUP-UNIPA_67390/2021 | EPI_ISL_13637913 | 6/4/2021 | 16 | M | Unknown | B.1.525 |
| hCoV-19/Italy/SIC-AOUP-UNIPA_67391/2021 | EPI_ISL_13637914 | 6/4/2021 | 24 | M | Syria | C.36.3 |
| hCoV-19/Italy/SIC-AOUP-UNIPA_67392/2021 | EPI_ISL_13637915 | 6/4/2021 | 19 | M | Unknown | B.1.525 |

| | | | | | | |
|---|---|---|---|---|---|---|
| hCoV-19/Italy/SIC-AOUP-UNIPA_67393/2021 | EPI_ISL_13637916 | 6/4/2021 | 21 | M | Senegal | B.1.525 |
| hCoV-19/Italy/SIC-AOUP-UNIPA_67394/2021 | EPI_ISL_13637917 | 6/4/2021 | 17 | M | Bangladesh | B.1.525 |
| hCoV-19/Italy/SIC-AOUP-UNIPA_67674/2021 | EPI_ISL_13637918 | 7/4/2021 | 17 | M | Guinea Conakry | B.1.525 |
| hCoV-19/Italy/SIC-AOUP-UNIPA_67675/2021 | EPI_ISL_13637919 | 7/4/2021 | 18 | M | Somalia | B.1.525 |
| hCoV-19/Italy/SIC-AOUP-UNIPA_67676/2021 | EPI_ISL_13637920 | 7/4/2021 | 16 | M | Ivory Coast | B.1.525 |
| hCoV-19/Italy/SIC-AOUP-UNIPA_67677/2021 | EPI_ISL_13637921 | 7/4/2021 | 21 | M | Sudan | B.1.525 |
| hCoV-19/Italy/SIC-AOUP-UNIPA_67678/2021 | EPI_ISL_13637922 | 7/4/2021 | 24 | M | Sudan | B.1.525 |
| hCoV-19/Italy/SIC-AOUP-UNIPA_67679/2021 | EPI_ISL_13637923 | 7/4/2021 | 17 | F | Ivory Coast | B.1.525 |
| hCoV-19/Italy/SIC-AOUP-UNIPA_68687/2021 | EPI_ISL_13637924 | 10/4/2021 | 40 | M | Eritrea | B.1.525 |
| hCoV-19/Italy/SIC-AOUP-UNIPA_68688/2021 | EPI_ISL_13637925 | 10/4/2021 | 21 | M | Ivory Coast | B.1.525 |
| hCoV-19/Italy/SIC-AOUP-UNIPA_68696/2021 | EPI_ISL_13637926 | 10/4/2021 | 17 | M | Somalia | B.1.525 |
| hCoV-19/Italy/SIC-AOUP-UNIPA_68697/2021 | EPI_ISL_13620695 | 10/4/2021 | 17 | M | Tunisia | B.1.1.7 |
| hCoV-19/Italy/SIC-AOUP-UNIPA_68698/2021 | EPI_ISL_13620685 | 10/4/2021 | 23 | M | Bangladesh | B.1.1.7 |
| hCoV-19/Italy/SIC-AOUP-UNIPA_68699/2021 | EPI_ISL_13637927 | 10/4/2021 | 17 | M | Somalia | B.1.525 |
| hCoV-19/Italy/SIC-AOUP-UNIPA_68701/2021 | EPI_ISL_13620696 | 10/4/2021 | 24 | M | Tunisia | B.1.1.7 |
| hCoV-19/Italy/SIC-AOUP-UNIPA_70333/2021 | EPI_ISL_6202105 | 16/4/2021 | 75 | M | Unknown | B.1.1.7 |
| hCoV-19/Italy/SIC-AOUP-UNIPA_75437/2021 | EPI_ISL_13620693 | 3/5/2021 | 46 | M | Indonesia | B.1.1.7 |
| hCoV-19/Italy/SIC-AOUP-UNIPA_75446/2021 | EPI_ISL_13620686 | 3/5/2021 | 38 | M | Tunisia | B.1.1.7 |
| hCoV-19/Italy/SIC-AOUP-UNIPA_75451/2021 | EPI_ISL_13637928 | 3/5/2021 | 26 | M | Sudan | B.1.525 |
| hCoV-19/Italy/SIC-AOUP-UNIPA_75452/2021 | EPI_ISL_13620683 | 3/5/2021 | 32 | M | Bangladesh | B.1.351 |
| hCoV-19/Italy/SIC-AOUP-UNIPA_75453/2021 | EPI_ISL_13637929 | 3/5/2021 | 22 | M | Bangladesh | B.1.525 |
| hCoV-19/Italy/SIC-AOUP-UNIPA_75454/2021 | EPI_ISL_6202082 | 3/5/2021 | 30 | M | Bangladesh | B.1.351.3 |

| | | | | | | |
|---|---|---|---|---|---|---|
| hCoV-19/Italy/SIC-AOUP-UNIPA_75455/2021 | EPI_ISL_13620678 | 3/5/2021 | 33 | M | Bangladesh | B.1.351.3 |
| hCoV-19/Italy/SIC-AOUP-UNIPA_75456/2021 | EPI_ISL_6202048 | 3/5/2021 | 26 | M | Morocco | B.1.525 |
| hCoV-19/Italy/SIC-AOUP-UNIPA_75457/2021 | EPI_ISL_13620684 | 3/5/2021 | 19 | M | Bangladesh | B.1.351 |
| hCoV-19/Italy/SIC-AOUP-UNIPA_75458/2021 | EPI_ISL_6202066 | 3/5/2021 | 14 | M | Burkina Faso | B.1.1.7 |
| hCoV-19/Italy/SIC-AOUP-UNIPA_75459/2021 | EPI_ISL_13620687 | 3/5/2021 | 18 | M | Ivory Coast | B.1.1.7 |
| hCoV-19/Italy/SIC-AOUP-UNIPA_75460/2021 | EPI_ISL_6202061 | 3/5/2021 | 17 | M | Ivory Coast | B.1.525 |
| hCoV-19/Italy/SIC-AOUP-UNIPA_75461/2021 | EPI_ISL_6202027 | 3/5/2021 | 29 | M | Ivory Coast | B.1.525 |
| hCoV-19/Italy/SIC-AOUP-UNIPA_75462/2021 | EPI_ISL_6202045 | 3/5/2021 | 13 | M | Ivory Coast | B.1.525 |
| hCoV-19/Italy/SIC-AOUP-UNIPA_75463/2021 | EPI_ISL_13637930 | 3/5/2021 | 20 | F | Ivory Coast | B.1.525 |
| hCoV-19/Italy/SIC-AOUP-UNIPA_75464/2021 | EPI_ISL_13620688 | 3/5/2021 | 4 | M | Guinea Conakry | B.1.1.7 |
| hCoV-19/Italy/SIC-AOUP-UNIPA_75465/2021 | EPI_ISL_6202040 | 3/5/2021 | 21 | F | Guinea Conakry | B.1.525 |
| hCoV-19/Italy/SIC-AOUP-UNIPA_76592/2021 | EPI_ISL_13637931 | 19/4/2021 | 25 | M | Ethiopia | B.1.525 |
| hCoV-19/Italy/SIC-AOUP-UNIPA_76596/2021 | EPI_ISL_13637932 | 7/5/2021 | 26 | M | Morocco | B.1.525 |
| hCoV-19/Italy/SIC-AOUP-UNIPA_76683/2021 | EPI_ISL_13620689 | 4/5/2021 | 18 | M | Tunisia | B.1.1.7 |
| hCoV-19/Italy/SIC-AOUP-UNIPA_78332/2021 | EPI_ISL_13637933 | 14/5/2021 | 25 | M | Morocco | B.1.525 |
| hCoV-19/Italy/SIC-AOUP-UNIPA_78333/2021 | EPI_ISL_13637934 | 14/5/2021 | 17 | M | Bangladesh | C.36.3 |
| hCoV-19/Italy/SIC-AOUP-UNIPA_78334/2021 | EPI_ISL_13637935 | 14/5/2021 | 25 | M | Egypt | C.36.3 |
| hCoV-19/Italy/SIC-AOUP-UNIPA_78913/2021 | EPI_ISL_13620701 | 28/4/2021 | 18 | M | Egypt | B.1.1.7 |
| hCoV-19/Italy/SIC-AOUP-UNIPA_78917/2021 | EPI_ISL_13620697 | 28/4/2021 | 31 | M | Egypt | B.1.1.7 |
| hCoV-19/Italy/SIC-AOUP-UNIPA_78918/2021 | EPI_ISL_13620694 | 28/4/2021 | 28 | M | Egypt | B.1.1.7 |
| hCoV-19/Italy/SIC-AOUP-UNIPA_78921/2021 | EPI_ISL_13622884 | 3/5/2021 | 18 | M | Burkina Faso | B.1.620 |
| hCoV-19/Italy/SIC-AOUP-UNIPA_78922/2021 | EPI_ISL_4050993 | 3/5/2021 | 19 | M | Burkina Faso | B.1.620 |

| | | | | | | |
|---|---|---|---|---|---|---|
| hCoV-19/Italy/SIC-AOUP-UNIPA_78923/2021 | EPI_ISL_13622885 | 3/5/2021 | 20 | M | Nigeria | B.1.620 |
| hCoV-19/Italy/SIC-AOUP-UNIPA_78924/2021 | EPI_ISL_13622886 | 3/5/2021 | 18 | M | Mali | B.1.620 |
| hCoV-19/Italy/SIC-AOUP-UNIPA_78925/2021 | EPI_ISL_13622887 | 3/5/2021 | 18 | M | Gambia | B.1.620 |
| hCoV-19/Italy/SIC-AOUP-UNIPA_78926/2021 | EPI_ISL_13622888 | 3/5/2021 | 18 | M | Guinea Conakry | B.1.620 |
| hCoV-19/Italy/SIC-AOUP-UNIPA_78927/2021 | EPI_ISL_13622889 | 3/5/2021 | 25 | M | Togo | B.1.620 |
| hCoV-19/Italy/SIC-AOUP-UNIPA_78928/2021 | EPI_ISL_13622890 | 3/5/2021 | 18 | M | Guinea Conakry | B.1.620 |
| hCoV-19/Italy/SIC-AOUP-UNIPA_78929/2021 | EPI_ISL_13622891 | 3/5/2021 | 29 | M | Burkina Faso | B.1.620 |
| hCoV-19/Italy/SIC-AOUP-UNIPA_78930/2021 | EPI_ISL_13622892 | 3/5/2021 | 25 | M | Burkina Faso | B.1.620 |
| hCoV-19/Italy/SIC-AOUP-UNIPA_78931/2021 | EPI_ISL_13622893 | 3/5/2021 | 18 | M | Guinea Conakry | B.1.620 |
| hCoV-19/Italy/SIC-AOUP-UNIPA_78932/2021 | EPI_ISL_13622894 | 3/5/2021 | 27 | M | Mali | B.1.620 |
| hCoV-19/Italy/SIC-AOUP-UNIPA_78933/2021 | EPI_ISL_13622895 | 3/5/2021 | 18 | M | Senegal | B.1.620 |
| hCoV-19/Italy/SIC-AOUP-UNIPA_78934/2021 | EPI_ISL_13622896 | 3/5/2021 | 19 | M | Cameroon | B.1.620 |
| hCoV-19/Italy/SIC-AOUP-UNIPA_78936/2021 | EPI_ISL_13622897 | 3/5/2021 | 18 | M | Gambia | B.1.620 |
| hCoV-19/Italy/SIC-AOUP-UNIPA_78937/2021 | EPI_ISL_13622898 | 3/5/2021 | 20 | M | Mali | B.1.620 |
| hCoV-19/Italy/SIC-AOUP-UNIPA_78938/2021 | EPI_ISL_13622899 | 3/5/2021 | 18 | M | Guinea Conakry | B.1.620 |
| hCoV-19/Italy/SIC-AOUP-UNIPA_78939/2021 | EPI_ISL_13622900 | 3/5/2021 | 18 | M | Guinea Conakry | B.1.620 |
| hCoV-19/Italy/SIC-AOUP-UNIPA_78940/2021 | EPI_ISL_13637936 | 3/5/2021 | 26 | M | Sudan | B.1.525 |
| hCoV-19/Italy/SIC-AOUP-UNIPA_78941/2021 | EPI_ISL_13622901 | 3/5/2021 | 25 | M | Burkina Faso | B.1.620 |
| hCoV-19/Italy/SIC-AOUP-UNIPA_78942/2021 | EPI_ISL_13622902 | 3/5/2021 | 23 | M | Mali | B.1.620 |
| hCoV-19/Italy/SIC-AOUP-UNIPA_78943/2021 | EPI_ISL_13622903 | 3/5/2021 | 23 | M | Mali | B.1.620 |
| hCoV-19/Italy/SIC-AOUP-UNIPA_78944/2021 | EPI_ISL_13622904 | 3/5/2021 | 21 | M | Gambia | B.1.620 |
| hCoV-19/Italy/SIC-AOUP-UNIPA_78945/2021 | EPI_ISL_13622905 | 3/5/2021 | 18 | M | Mali | B.1.620 |

| | | | | | | |
|---|---|---|---|---|---|---|
| hCoV-19/Italy/SIC-AOUP-UNIPA_78946/2021 | EPI_ISL_13622906 | 3/5/2021 | 18 | M | Burkina Faso | B.1.620 |
| hCoV-19/Italy/SIC-AOUP-UNIPA_78947/2021 | EPI_ISL_13622907 | 3/5/2021 | 18 | M | Ivory Coast | B.1.620 |
| hCoV-19/Italy/SIC-AOUP-UNIPA_78948/2021 | EPI_ISL_13622908 | 3/5/2021 | 18 | M | Guinea Conakry | B.1.620 |
| hCoV-19/Italy/SIC-AOUP-UNIPA_78949/2021 | EPI_ISL_13622909 | 3/5/2021 | 31 | M | Cameroon | B.1.620 |
| hCoV-19/Italy/SIC-AOUP-UNIPA_78950/2021 | EPI_ISL_13622910 | 3/5/2021 | 18 | M | Burkina Faso | B.1.620 |
| hCoV-19/Italy/SIC-AOUP-UNIPA_78951/2021 | EPI_ISL_13622911 | 3/5/2021 | 26 | M | Ghana | B.1.620 |
| hCoV-19/Italy/SIC-AOUP-UNIPA_78952/2021 | EPI_ISL_13622912 | 3/5/2021 | 22 | M | Burkina Faso | B.1.620 |
| hCoV-19/Italy/SIC-AOUP-UNIPA_78953/2021 | EPI_ISL_13622913 | 3/5/2021 | 20 | M | Burkina Faso | B.1.620 |
| hCoV-19/Italy/SIC-AOUP-UNIPA_78954/2021 | EPI_ISL_13622914 | 3/5/2021 | 24 | M | Burkina Faso | B.1.620 |
| hCoV-19/Italy/SIC-AOUP-UNIPA_78955/2021 | EPI_ISL_13622915 | 3/5/2021 | 18 | M | Mali | B.1.620 |
| hCoV-19/Italy/SIC-AOUP-UNIPA_78956/2021 | EPI_ISL_13622916 | 3/5/2021 | 28 | F | Mali | B.1.620 |
| hCoV-19/Italy/SIC-AOUP-UNIPA_78957/2021 | EPI_ISL_13622917 | 3/5/2021 | 19 | M | Mali | B.1.620 |
| hCoV-19/Italy/SIC-AOUP-UNIPA_78958/2021 | EPI_ISL_6202019 | 3/5/2021 | 18 | M | Ghana | B.1.620 |
| hCoV-19/Italy/SIC-AOUP-UNIPA_78959/2021 | EPI_ISL_13622918 | 3/5/2021 | 31 | M | Niger | B.1.620 |
| hCoV-19/Italy/SIC-AOUP-UNIPA_78960/2021 | EPI_ISL_13622919 | 3/5/2021 | 22 | M | Cameroon | B.1.620 |
| hCoV-19/Italy/SIC-AOUP-UNIPA_78961/2021 | EPI_ISL_13622920 | 3/5/2021 | 18 | M | Cameroon | B.1.620 |
| hCoV-19/Italy/SIC-AOUP-UNIPA_78962/2021 | EPI_ISL_13622921 | 3/5/2021 | 22 | M | Cameroon | B.1.620 |
| hCoV-19/Italy/SIC-AOUP-UNIPA_78965/2021 | EPI_ISL_13620698 | 8/5/2021 | 19 | M | Egypt | B.1.1.7 |
| hCoV-19/Italy/SIC-AOUP-UNIPA_78966/2021 | EPI_ISL_13620699 | 8/5/2021 | 40 | M | Egypt | B.1.1.7 |
| hCoV-19/Italy/SIC-AOUP-UNIPA_78967/2021 | EPI_ISL_13622922 | 8/5/2021 | 25 | M | Togo | B.1.620 |
| hCoV-19/Italy/SIC-AOUP-UNIPA_78968/2021 | EPI_ISL_13622923 | 8/5/2021 | 44 | M | Senegal | B.1.620 |
| hCoV-19/Italy/SIC-AOUP-UNIPA_78970/2021 | EPI_ISL_13622924 | 8/5/2021 | 33 | M | Kenya | B.1.620 |

| | | | | | | |
|---|---|---|---|---|---|---|
| hCoV-19/Italy/SIC-AOUP-UNIPA_78971/2021 | EPI_ISL_13620681 | 9/5/2021 | 27 | M | Libya | B.1.1.7 |
| hCoV-19/Italy/SIC-AOUP-UNIPA_78972/2021 | EPI_ISL_13620680 | 9/5/2021 | 23 | M | Gambia | B.1.1.7 |
| hCoV-19/Italy/SIC-AOUP-UNIPA_78973/2021 | EPI_ISL_13622925 | 11/5/2021 | 19 | M | Gambia | B.1.620 |
| hCoV-19/Italy/SIC-AOUP-UNIPA_78974/2021 | EPI_ISL_13622926 | 11/5/2021 | 26 | M | Cameroon | B.1.620 |
| hCoV-19/Italy/SIC-AOUP-UNIPA_78975/2021 | EPI_ISL_13622927 | 11/5/2021 | 25 | M | Sudan | B.1.620 |
| hCoV-19/Italy/SIC-AOUP-UNIPA_78976/2021 | EPI_ISL_4050991 | 11/5/2021 | 22 | M | Sudan | B.1.620 |
| hCoV-19/Italy/SIC-AOUP-UNIPA_78977/2021 | EPI_ISL_13622928 | 11/5/2021 | 18 | M | Ghana | B.1.620 |
| hCoV-19/Italy/SIC-AOUP-UNIPA_78978/2021 | EPI_ISL_13622929 | 11/5/2021 | 18 | M | Ghana | B.1.620 |
| hCoV-19/Italy/SIC-AOUP-UNIPA_78979/2021 | EPI_ISL_13622930 | 11/5/2021 | 20 | M | Ghana | B.1.620 |
| hCoV-19/Italy/SIC-AOUP-UNIPA_80222/2021 | EPI_ISL_13637937 | 26/5/2021 | 23 | M | Unknown | C.36.3 |
| hCoV-19/Italy/SIC-AOUP-UNIPA_80542/2021 | EPI_ISL_13637938 | 29/5/2021 | 23 | M | Bangladesh | P.1.1 |
| hCoV-19/Italy/SIC-AOUP-UNIPA_80903/2021 | EPI_ISL_13666877 | 29/5/2021 | 21 | M | Bangladesh | B.1.617.2 |
| hCoV-19/Italy/SIC-AOUP-UNIPA_80904/2021 | EPI_ISL_13666878 | 29/5/2021 | 21 | M | Bangladesh | B.1.617.2 |
| hCoV-19/Italy/SIC-AOUP-UNIPA_80905/2021 | EPI_ISL_13666879 | 29/5/2021 | 17 | M | Bangladesh | B.1.617.2 |
| hCoV-19/Italy/SIC-AOUP-UNIPA_80906/2021 | EPI_ISL_13666880 | 29/5/2021 | 18 | M | Bangladesh | B.1.617.2 |
| hCoV-19/Italy/SIC-AOUP-UNIPA_80907/2021 | EPI_ISL_13666881 | 29/5/2021 | 25 | M | Bangladesh | B.1.617.2 |
| hCoV-19/Italy/SIC-AOUP-UNIPA_80908/2021 | EPI_ISL_13666882 | 29/5/2021 | 18 | M | Bangladesh | B.1.617.2 |
| hCoV-19/Italy/SIC-AOUP-UNIPA_80909/2021 | EPI_ISL_13666883 | 29/5/2021 | 35 | M | Bangladesh | B.1.617.2 |
| hCoV-19/Italy/SIC-AOUP-UNIPA_80910/2021 | EPI_ISL_13666884 | 29/5/2021 | 35 | M | Bangladesh | B.1.617.2 |
| hCoV-19/Italy/SIC-AOUP-UNIPA_80911/2021 | EPI_ISL_13666885 | 29/5/2021 | 32 | M | Bangladesh | B.1.617.2 |
| hCoV-19/Italy/SIC-AOUP-UNIPA_80912/2021 | EPI_ISL_13666886 | 29/5/2021 | 18 | M | Bangladesh | B.1.617.2 |
| hCoV-19/Italy/SIC-AOUP-UNIPA_80913/2021 | EPI_ISL_13622931 | 29/5/2021 | 30 | M | Morocco | B.1.620 |

| | | | | | | |
|---|---|---|---|---|---|---|
| hCoV-19/Italy/SIC-AOUP-UNIPA_81966/2021 | EPI_ISL_13637939 | 12/6/2021 | 20 | M | Guinea Conakry | B.1.525 |
| hCoV-19/Italy/SIC-AOUP-UNIPA_81971/2021 | EPI_ISL_13666887 | 12/6/2021 | 17 | M | Bangladesh | B.1.617.2 |
| hCoV-19/Italy/SIC-AOUP-UNIPA_81972/2021 | EPI_ISL_6201992 | 12/6/2021 | 17 | M | Bangladesh | B.1.617.2 |
| hCoV-19/Italy/SIC-AOUP-UNIPA_81973/2021 | EPI_ISL_2650009 | 12/6/2021 | 17 | M | Bangladesh | B.1.617.2 |
| hCoV-19/Italy/SIC-AOUP-UNIPA_82042/2021 | EPI_ISL_13620692 | 14/6/2021 | 14 | F | Tunisia | B.1.1.7 |
| hCoV-19/Italy/SIC-AOUP-UNIPA_82043/2021 | EPI_ISL_13620690 | 14/6/2021 | 9 | M | Tunisia | B.1.1.7 |
| hCoV-19/Italy/SIC-AOUP-UNIPA_82044/2021 | EPI_ISL_13620702 | 14/6/2021 | 8 | M | Tunisia | B.1.1.7 |
| hCoV-19/Italy/SIC-AOUP-UNIPA_82045/2021 | EPI_ISL_2765561 | 14/6/2021 | 28 | M | Egypt | C.36.3 |
| hCoV-19/Italy/SIC-AOUP-UNIPA_82046/2021 | EPI_ISL_13620691 | 14/6/2021 | 40 | F | Algeria | B.1.1.7 |
| hCoV-19/Italy/SIC-AOUP-UNIPA_82240/2021 | EPI_ISL_2650444 | 13/6/2021 | 24 | M | Bangladesh | B.1.617.2 |
| hCoV-19/Italy/SIC-AOUP-UNIPA_82241/2021 | EPI_ISL_2650445 | 13/6/2021 | 19 | M | Bangladesh | B.1.617.2 |
| hCoV-19/Italy/SIC-AOUP-UNIPA_82242/2021 | EPI_ISL_2650449 | 13/6/2021 | 18 | M | Bangladesh | B.1.617.2 |
| hCoV-19/Italy/SIC-AOUP-UNIPA_82243/2021 | EPI_ISL_2650450 | 13/6/2021 | 19 | M | Bangladesh | B.1.617.2 |
| hCoV-19/Italy/SIC-AOUP-UNIPA_82244/2021 | EPI_ISL_2650448 | 13/6/2021 | 24 | M | Bangladesh | B.1.617.2 |
| hCoV-19/Italy/SIC-AOUP-UNIPA_82245/2021 | EPI_ISL_2650446 | 13/6/2021 | 16 | M | Bangladesh | B.1.617.2 |
| hCoV-19/Italy/SIC-AOUP-UNIPA_82246/2021 | EPI_ISL_2650447 | 13/6/2021 | 21 | M | Bangladesh | B.1.617.2 |
| hCoV-19/Italy/SIC-AOUP-UNIPA_82247/2021 | EPI_ISL_2650443 | 13/6/2021 | 22 | M | Bangladesh | B.1.617.2 |
| hCoV-19/Italy/SIC-AOUP-UNIPA_82248/2021 | EPI_ISL_2650441 | 13/6/2021 | 18 | M | Bangladesh | B.1.617.2 |
| hCoV-19/Italy/SIC-AOUP-UNIPA_82249/2021 | EPI_ISL_2650440 | 13/6/2021 | 41 | M | Bangladesh | B.1.617.2 |
| hCoV-19/Italy/SIC-AOUP-UNIPA_82250/2021 | EPI_ISL_2650439 | 13/6/2021 | 18 | M | Bangladesh | B.1.617.2 |
| hCoV-19/Italy/SIC-AOUP-UNIPA_82251/2021 | EPI_ISL_2650433 | 13/6/2021 | 31 | M | Bangladesh | B.1.617.2 |
| hCoV-19/Italy/SIC-AOUP-UNIPA_82252/2021 | EPI_ISL_2650008 | 13/6/2021 | 27 | M | Morocco | B.1.525 |

| | | | | | | |
|---|---|---|---|---|---|---|
| hCoV-19/Italy/SIC-AOUP-UNIPA_82253/2021 | EPI_ISL_3915319 | 13/6/2021 | 35 | M | Sudan | B.1.525 |
| hCoV-19/Italy/SIC-AOUP-UNIPA_82254/2021 | EPI_ISL_2650442 | 13/6/2021 | 26 | M | Cameroon | B.1.525 |
| hCoV-19/Italy/SIC-AOUP-UNIPA_82255/2021 | EPI_ISL_3915320 | 13/6/2021 | 20 | M | Sudan | B.1.525 |
| hCoV-19/Italy/SIC-AOUP-UNIPA_82256/2021 | EPI_ISL_3915321 | 13/6/2021 | 15 | M | Cameroon | B.1.525 |
| hCoV-19/Italy/SIC-AOUP-UNIPA_82258/2021 | EPI_ISL_3915317 | 13/6/2021 | 22 | M | Egypt | B.1.525 |
| hCoV-19/Italy/SIC-AOUP-UNIPA_82259/2021 | EPI_ISL_3915316 | 13/6/2021 | 25 | M | Sudan | B.1.525 |
| hCoV-19/Italy/SIC-AOUP-UNIPA_82260/2021 | EPI_ISL_2650434 | 13/6/2021 | 26 | M | Yemen | B.1.525 |
| hCoV-19/Italy/SIC-AOUP-UNIPA_82261/2021 | EPI_ISL_3915315 | 13/6/2021 | 15 | M | Sudan | B.1.525 |
| hCoV-19/Italy/SIC-AOUP-UNIPA_82262/2021 | EPI_ISL_3915318 | 13/6/2021 | 19 | M | Sudan | B.1.525 |
| hCoV-19/Italy/SIC-AOUP-UNIPA_82263/2021 | EPI_ISL_3915314 | 13/6/2021 | 21 | M | Sudan | B.1.525 |
| hCoV-19/Italy/SIC-AOUP-UNIPA_82264/2021 | EPI_ISL_3915312 | 13/6/2021 | 18 | M | Sudan | B.1.525 |
| hCoV-19/Italy/SIC-AOUP-UNIPA_82266/2021 | EPI_ISL_3915313 | 13/6/2021 | 28 | M | Morocco | B.1.525 |
| hCoV-19/Italy/SIC-AOUP-UNIPA_82267/2021 | EPI_ISL_3915311 | 13/6/2021 | 29 | M | Morocco | B.1.525 |
| hCoV-19/Italy/SIC-AOUP-UNIPA_82268/2021 | EPI_ISL_3915310 | 13/6/2021 | 28 | M | Morocco | B.1.525 |
| hCoV-19/Italy/SIC-AOUP-UNIPA_82269/2021 | EPI_ISL_2650438 | 15/6/2021 | 28 | M | Egypt | C.36.3 |
| hCoV-19/Italy/SIC-AOUP-UNIPA_82270/2021 | EPI_ISL_2650437 | 15/6/2021 | 33 | M | Egypt | B.1.617.2 |
| hCoV-19/Italy/SIC-AOUP-UNIPA_82271/2021 | EPI_ISL_2650435 | 15/6/2021 | 26 | M | Sudan | B.1.617.2 |
| hCoV-19/Italy/SIC-AOUP-UNIPA_82272/2021 | EPI_ISL_3915308 | 15/6/2021 | 23 | M | Bangladesh | B.1.525 |
| hCoV-19/Italy/SIC-AOUP-UNIPA_82273/2021 | EPI_ISL_2650436 | 15/6/2021 | 19 | M | Ivory Coast | B.1.1.7 |
| hCoV-19/Italy/SIC-AOUP-UNIPA_82274/2021 | EPI_ISL_3915309 | 15/6/2021 | 26 | M | Ivory Coast | B.1.1.7 |
| hCoV-19/Italy/SIC-AOUP-UNIPA_82533/2021 | EPI_ISL_3915307 | 17/6/2021 | 25 | M | Tunisia | AY.122 |
| hCoV-19/Italy/SIC-AOUP-UNIPA_82534/2021 | EPI_ISL_3915306 | 17/6/2021 | 14 | M | Tunisia | B.1.617.2 |

| | | | | | | |
|---|---|---|---|---|---|---|
| hCoV-19/Italy/SIC-AOUP-UNIPA_82535/2021 | EPI_ISL_3915304 | 17/6/2021 | 46 | M | Egypt | C.36.3 |
| hCoV-19/Italy/SIC-AOUP-UNIPA_82536/2021 | EPI_ISL_3915300 | 16/6/2021 | 26 | M | Ethiopia | B.1.525 |
| hCoV-19/Italy/SIC-AOUP-UNIPA_82537/2021 | EPI_ISL_3915303 | 16/6/2021 | 17 | M | Egypt | C.36.3 |
| hCoV-19/Italy/SIC-AOUP-UNIPA_82538/2021 | EPI_ISL_3915302 | 17/6/2021 | 32 | M | Morocco | C.36.3 |
| hCoV-19/Italy/SIC-AOUP-UNIPA_82540/2021 | EPI_ISL_3915297 | 15/6/2021 | 19 | M | Tunisia | AY.122 |
| hCoV-19/Italy/SIC-AOUP-UNIPA_82541/2021 | EPI_ISL_6202002 | 15/6/2021 | 17 | M | Tunisia | AY.122 |
| hCoV-19/Italy/SIC-AOUP-UNIPA_82542/2021 | EPI_ISL_3915299 | 19/6/2021 | 25 | M | Sudan | B.1.525 |
| hCoV-19/Italy/SIC-AOUP-UNIPA_82656/2021 | EPI_ISL_3915298 | 19/6/2021 | 18 | M | Tunisia | B.1.525 |
| hCoV-19/Italy/SIC-AOUP-UNIPA_82657/2021 | EPI_ISL_3915295 | 19/6/2021 | 30 | M | Ivory Coast | B.1.1.7 |
| hCoV-19/Italy/SIC-AOUP-UNIPA_82658/2021 | EPI_ISL_3915293 | 19/6/2021 | 22 | M | Tunisia | B.1.617.2 |
| hCoV-19/Italy/SIC-AOUP-UNIPA_82659/2021 | EPI_ISL_3915296 | 19/6/2021 | 33 | M | Algeria | B.1.525 |
| hCoV-19/Italy/SIC-AOUP-UNIPA_82660/2021 | EPI_ISL_3915294 | 19/6/2021 | 27 | M | Egypt | C.36.3 |
| hCoV-19/Italy/SIC-AOUP-UNIPA_82661/2021 | EPI_ISL_3915292 | 19/6/2021 | 23 | M | Egypt | C.36.3 |
| hCoV-19/Italy/SIC-AOUP-UNIPA_82662/2021 | EPI_ISL_13666888 | 20/6/2021 | 21 | M | Bangladesh | B.1.617.2 |
| hCoV-19/Italy/SIC-AOUP-UNIPA_82663/2021 | EPI_ISL_13666889 | 20/6/2021 | 14 | M | Tunisia | AY.122 |
| hCoV-19/Italy/SIC-AOUP-UNIPA_82664/2021 | EPI_ISL_3915290 | 20/6/2021 | 14 | M | Tunisia | B.1.617.2 |
| hCoV-19/Italy/SIC-AOUP-UNIPA_82665/2021 | EPI_ISL_3915291 | 20/6/2021 | 15 | M | Tunisia | AY.122 |
| hCoV-19/Italy/SIC-AOUP-UNIPA_82666/2021 | EPI_ISL_3915287 | 20/6/2021 | 36 | M | Ivory Coast | B.1.525 |
| hCoV-19/Italy/SIC-AOUP-UNIPA_82667/2021 | EPI_ISL_3915288 | 19/6/2021 | 22 | M | Egypt | C.36.3 |
| hCoV-19/Italy/SIC-AOUP-UNIPA_82699/2021 | EPI_ISL_2694865 | 20/6/2021 | 17 | M | Bangladesh | B.1.617.2 |
| hCoV-19/Italy/SIC-AOUP-UNIPA_82812/2021 | EPI_ISL_6201984 | 21/6/2021 | 20 | M | Iran | AY.126 |
| hCoV-19/Italy/SIC-AOUP-UNIPA_82818/2021 | EPI_ISL_3915289 | 21/6/2021 | 27 | M | Afghanistan | AY.126 |

| | | | | | | |
|---|---|---|---|---|---|---|
| hCoV-19/Italy/SIC-AOUP-UNIPA_82819/2021 | EPI_ISL_3915286 | 21/6/2021 | 23 | M | Afghanistan | AY.126 |
| hCoV-19/Italy/SIC-AOUP-UNIPA_82820/2021 | EPI_ISL_4050992 | 21/6/2021 | 26 | F | Afghanistan | AY.126 |
| hCoV-19/Italy/SIC-AOUP-UNIPA_82821/2021 | EPI_ISL_4050990 | 21/6/2021 | 26 | M | Iran | AY.126 |
| hCoV-19/Italy/SIC-AOUP-UNIPA_82822/2021 | EPI_ISL_3915284 | 21/6/2021 | 26 | M | Egypt | B.1.617.2 |
| hCoV-19/Italy/SIC-AOUP-UNIPA_82823/2021 | EPI_ISL_3915285 | 21/6/2021 | 19 | M | Guinea Conakry | B.1.617.2 |
| hCoV-19/Italy/SIC-AOUP-UNIPA_82827/2021 | EPI_ISL_4050989 | 21/6/2021 | 25 | M | Bangladesh | B.1.617.2 |
| hCoV-19/Italy/SIC-AOUP-UNIPA_82893/2021 | EPI_ISL_6202007 | 22/6/2021 | 34 | M | Egypt | B.1.1.7 |
| hCoV-19/Italy/SIC-AOUP-UNIPA_83018/2021 | EPI_ISL_2861822 | 24/6/2021 | 17 | M | Tunisia | B.1.617.2 |
| hCoV-19/Italy/SIC-AOUP-UNIPA_83023/2021 | EPI_ISL_2861820 | 25/6/2021 | 17 | M | Tunisia | B.1.617.2 |
| hCoV-19/Italy/SIC-AOUP-UNIPA_83024/2021 | EPI_ISL_2886182 | 25/6/2021 | 15 | M | Tunisia | B.1.617.2 |
| hCoV-19/Italy/SIC-AOUP-UNIPA_83027/2021 | EPI_ISL_6201972 | 25/6/2021 | 16 | M | Tunisia | B.1.617.2 |
| hCoV-19/Italy/SIC-AOUP-UNIPA_83028/2021 | EPI_ISL_2861819 | 25/6/2021 | 17 | M | Tunisia | B.1.617.2 |
| hCoV-19/Italy/SIC-AOUP-UNIPA_83029/2021 | EPI_ISL_6201965 | 25/6/2021 | 15 | M | Tunisia | B.1.617.2 |
| hCoV-19/Italy/SIC-AOUP-UNIPA_83033/2021 | EPI_ISL_6201940 | 25/6/2021 | 16 | M | Tunisia | B.1.617.2 |
| hCoV-19/Italy/SIC-AOUP-UNIPA_83034/2021 | EPI_ISL_2886199 | 25/6/2021 | 15 | M | Tunisia | B.1.617.2 |
| hCoV-19/Italy/SIC-AOUP-UNIPA_83035/2021 | EPI_ISL_6201954 | 25/6/2021 | 17 | M | Morocco | B.1.617.2 |
| hCoV-19/Italy/SIC-AOUP-UNIPA_83193/2021 | EPI_ISL_2820500 | 30/6/2021 | 21 | M | Egypt | C.38 |
| hCoV-19/Italy/SIC-AOUP-UNIPA_83194/2021 | EPI_ISL_2820499 | 28/6/2021 | 22 | M | Egypt | C.38 |
| hCoV-19/Italy/SIC-AOUP-UNIPA_83222/2021 | EPI_ISL_2886198 | 29/6/2021 | 24 | M | Morocco | B.1.525 |
| hCoV-19/Italy/SIC-AOUP-UNIPA_83223/2021 | EPI_ISL_2820495 | 29/6/2021 | 18 | M | Egypt | C.36.3 |
| hCoV-19/Italy/SIC-AOUP-UNIPA_83224/2021 | EPI_ISL_2820489 | 29/6/2021 | 22 | M | Egypt | C.36.3 |
| hCoV-19/Italy/SIC-AOUP-UNIPA_83225/2021 | EPI_ISL_2820494 | 29/6/2021 | 18 | M | Egypt | B.1.525 |

| | | | | | | |
|---|---|---|---|---|---|---|
| hCoV-19/Italy/SIC-AOUP-UNIPA_83227/2021 | EPI_ISL_2820493 | 29/6/2021 | 33 | M | Egypt | C.36.3 |
| hCoV-19/Italy/SIC-AOUP-UNIPA_83228/2021 | EPI_ISL_2886200 | 29/6/2021 | 27 | M | Morocco | B.1.525 |
| hCoV-19/Italy/SIC-AOUP-UNIPA_83294/2021 | EPI_ISL_2861815 | 29/6/2021 | 17 | M | Eritrea | B.1.617.2 |
| hCoV-19/Italy/SIC-AOUP-UNIPA_83295/2021 | EPI_ISL_2886186 | 29/6/2021 | 16 | M | Tunisia | B.1.617.2 |
| hCoV-19/Italy/SIC-AOUP-UNIPA_83296/2021 | EPI_ISL_2861817 | 29/6/2021 | 14 | M | Tunisia | B.1.617.2 |
| hCoV-19/Italy/SIC-AOUP-UNIPA_83300/2021 | EPI_ISL_2886189 | 29/6/2021 | 16 | M | Tunisia | B.1.617.2 |
| hCoV-19/Italy/SIC-AOUP-UNIPA_83302/2021 | EPI_ISL_6201945 | 29/6/2021 | 17 | M | Tunisia | B.1.617.2 |
| hCoV-19/Italy/SIC-AOUP-UNIPA_83303/2021 | EPI_ISL_6201928 | 29/6/2021 | 17 | M | Egypt | B.1.617.2 |
| hCoV-19/Italy/SIC-AOUP-UNIPA_83308/2021 | EPI_ISL_6201920 | 29/6/2021 | 16 | M | Morocco | B.1.617.2 |
| hCoV-19/Italy/SIC-AOUP-UNIPA_83315/2021 | EPI_ISL_13666890 | 29/6/2021 | 16 | M | Tunisia | B.1.617.2 |
| hCoV-19/Italy/SIC-AOUP-UNIPA_83333/2021 | EPI_ISL_6201902 | 30/6/2021 | 21 | M | Iran | AY.5 |
| hCoV-19/Italy/SIC-AOUP-UNIPA_83354/2021 | EPI_ISL_6201910 | 30/6/2021 | 15 | M | Tunisia | B.1.617.2 |
| hCoV-19/Italy/SIC-AOUP-UNIPA_83417/2021 | EPI_ISL_13695010 | 30/6/2021 | 35 | F | Ivory Coast | B.1.525 |
| hCoV-19/Italy/SIC-AOUP-UNIPA_83418/2021 | EPI_ISL_13666891 | 1/7/2021 | 21 | M | Egypt | B.1.617.2 |
| hCoV-19/Italy/SIC-AOUP-UNIPA_83648/2021 | EPI_ISL_6201865 | 30/6/2021 | 34 | M | Unknown | B.1.1.7 |
| hCoV-19/Italy/SIC-AOUP-UNIPA_83734/2021 | EPI_ISL_6201873 | 27/6/2021 | 18 | M | Ivory Coast | B.1.617.2 |
| hCoV-19/Italy/SIC-AOUP-UNIPA_83736/2021 | EPI_ISL_13666892 | 27/6/2021 | 24 | M | Tunisia | B.1.617.2 |
| hCoV-19/Italy/SIC-AOUP-UNIPA_83771/2021 | EPI_ISL_2975192 | 3/7/2021 | 35 | M | Tunisia | AY.122 |
| hCoV-19/Italy/SIC-AOUP-UNIPA_83772/2021 | EPI_ISL_13695011 | 4/7/2021 | 31 | M | Tunisia | AY.122 |
| hCoV-19/Italy/SIC-AOUP-UNIPA_83773/2021 | EPI_ISL_13695012 | 4/7/2021 | 23 | M | Tunisia | AY.122 |
| hCoV-19/Italy/SIC-AOUP-UNIPA_83774/2021 | EPI_ISL_13666893 | 4/7/2021 | 19 | M | Tunisia | AY.122 |
| hCoV-19/Italy/SIC-AOUP-UNIPA_83775/2021 | EPI_ISL_6201895 | 4/7/2021 | 17 | M | Tunisia | AY.122 |

| | | | | | | |
|---|---|---|---|---|---|---|
| hCoV-19/Italy/SIC-AOUP-UNIPA_83776/2021 | EPI_ISL_13666894 | 4/7/2021 | 23 | M | Tunisia | AY.122 |
| hCoV-19/Italy/SIC-AOUP-UNIPA_83777/2021 | EPI_ISL_13666895 | 4/7/2021 | 23 | M | Tunisia | AY.122 |
| hCoV-19/Italy/SIC-AOUP-UNIPA_83778/2021 | EPI_ISL_6201827 | 4/7/2021 | 27 | M | Tunisia | AY.122 |
| hCoV-19/Italy/SIC-AOUP-UNIPA_83779/2021 | EPI_ISL_13666896 | 4/7/2021 | 17 | M | Tunisia | AY.122 |
| hCoV-19/Italy/SIC-AOUP-UNIPA_83780/2021 | EPI_ISL_13666897 | 4/7/2021 | 16 | M | Tunisia | AY.122 |
| hCoV-19/Italy/SIC-AOUP-UNIPA_83781/2021 | EPI_ISL_13666898 | 4/7/2021 | 20 | M | Tunisia | AY.122 |
| hCoV-19/Italy/SIC-AOUP-UNIPA_83782/2021 | EPI_ISL_13666899 | 4/7/2021 | 16 | M | Tunisia | AY.122 |
| hCoV-19/Italy/SIC-AOUP-UNIPA_83783/2021 | EPI_ISL_13666900 | 4/7/2021 | 23 | M | Tunisia | AY.122 |
| hCoV-19/Italy/SIC-AOUP-UNIPA_83784/2021 | EPI_ISL_13666901 | 4/7/2021 | 21 | M | Tunisia | AY.122 |
| hCoV-19/Italy/SIC-AOUP-UNIPA_83785/2021 | EPI_ISL_13666902 | 4/7/2021 | 14 | M | Tunisia | AY.122 |
| hCoV-19/Italy/SIC-AOUP-UNIPA_83786/2021 | EPI_ISL_13666903 | 4/7/2021 | 20 | M | Tunisia | AY.122 |
| hCoV-19/Italy/SIC-AOUP-UNIPA_83902/2021 | EPI_ISL_13666904 | 7/7/2021 | 16 | M | Tunisia | B.1.617.2 |
| hCoV-19/Italy/SIC-AOUP-UNIPA_83917/2021 | EPI_ISL_6201856 | 8/7/2021 | 37 | M | Tunisia | AY.122 |
| hCoV-19/Italy/SIC-AOUP-UNIPA_84503/2021 | EPI_ISL_6201847 | 12/7/2021 | 17 | M | Bangladesh | B.1.617.2 |
| hCoV-19/Italy/SIC-AOUP-UNIPA_84504/2021 | EPI_ISL_6201883 | 12/7/2021 | 24 | M | Bangladesh | B.1.617.2 |
| hCoV-19/Italy/SIC-AOUP-UNIPA_84505/2021 | EPI_ISL_13666905 | 17/7/2021 | 17 | M | Tunisia | B.1.617.2 |
| hCoV-19/Italy/SIC-AOUP-UNIPA_84597/2021 | EPI_ISL_3128470 | 16/7/2021 | 37 | M | Bangladesh | B.1.617.2 |
| hCoV-19/Italy/SIC-AOUP-UNIPA_84598/2021 | EPI_ISL_3128468 | 12/7/2021 | 4 | F | Syria | B.1.617.2 |
| hCoV-19/Italy/SIC-AOUP-UNIPA_84931/2021 | EPI_ISL_13666906 | 16/7/2021 | 17 | M | Egypt | B.1.617.2 |
| hCoV-19/Italy/SIC-AOUP-UNIPA_84932/2021 | EPI_ISL_6201838 | 16/7/2021 | 17 | M | Egypt | B.1.617.2 |
| hCoV-19/Italy/SIC-AOUP-UNIPA_84945/2021 | EPI_ISL_6201815 | 19/7/2021 | 20 | M | Unknown | AY.122 |
| hCoV-19/Italy/SIC-AOUP-UNIPA_85095/2021 | EPI_ISL_6201803 | 16/7/2021 | 29 | M | Ghana | B.1.617.2 |

| | | | | | | |
|---|---|---|---|---|---|---|
| hCoV-19/Italy/SIC-AOUP-UNIPA_85255/2021 | EPI_ISL_6201796 | 16/7/2021 | 22 | M | Gambia | B.1.617.2 |
| hCoV-19/Italy/SIC-AOUP-UNIPA_85258/2021 | EPI_ISL_6201783 | 19/7/2021 | 22 | F | Tunisia | AY.122 |
| hCoV-19/Italy/SIC-AOUP-UNIPA_85432/2021 | EPI_ISL_6201759 | 19/7/2021 | | M | Unknown | AY.122 |
| hCoV-19/Italy/SIC-AOUP-UNIPA_85689/2021 | EPI_ISL_6201763 | 22/7/2021 | 28 | M | Tunisia | AY.122 |
| hCoV-19/Italy/SIC-AOUP-UNIPA_85855/2021 | EPI_ISL_13666907 | 22/7/2021 | 34 | F | Tunisia | B.1.617.2 |
| hCoV-19/Italy/SIC-AOUP-UNIPA_85856/2021 | EPI_ISL_13666908 | 23/7/2021 | 17 | M | Tunisia | B.1.617.2 |
| hCoV-19/Italy/SIC-AOUP-UNIPA_85857/2021 | EPI_ISL_13666909 | 23/7/2021 | 17 | M | Egypt | B.1.617.2 |
| hCoV-19/Italy/SIC-AOUP-UNIPA_85906/2021 | EPI_ISL_6201754 | 24/7/2021 | 22 | M | Ivory Coast | AY.122 |
| hCoV-19/Italy/SIC-AOUP-UNIPA_85907/2021 | EPI_ISL_13666910 | 24/7/2021 | 21 | M | Tunisia | AY.122 |
| hCoV-19/Italy/SIC-AOUP-UNIPA_85908/2021 | EPI_ISL_13666911 | 24/7/2021 | 22 | F | Tunisia | AY.122 |
| hCoV-19/Italy/SIC-AOUP-UNIPA_85909/2021 | EPI_ISL_13666912 | 24/7/2021 | 22 | F | Tunisia | AY.122 |
| hCoV-19/Italy/SIC-AOUP-UNIPA_85910/2021 | EPI_ISL_13666913 | 24/7/2021 | 27 | M | Tunisia | AY.122 |
| hCoV-19/Italy/SIC-AOUP-UNIPA_85912/2021 | EPI_ISL_13666914 | 24/7/2021 | 23 | F | Tunisia | AY.122 |
| hCoV-19/Italy/SIC-AOUP-UNIPA_85926/2021 | EPI_ISL_13666915 | 23/7/2021 | 17 | M | Bangladesh | B.1.617.2 |
| hCoV-19/Italy/SIC-AOUP-UNIPA_86096/2021 | EPI_ISL_3280872 | 27/7/2021 | 41 | M | Tunisia | AY.122 |
| hCoV-19/Italy/SIC-AOUP-UNIPA_86108/2021 | EPI_ISL_13666916 | 25/7/2021 | 3 | M | Tunisia | B.1.617.2 |
| hCoV-19/Italy/SIC-AOUP-UNIPA_86194/2021 | EPI_ISL_13666917 | 25/7/2021 | 26 | M | Bangladesh | B.1.617.2 |
| hCoV-19/Italy/SIC-AOUP-UNIPA_86221/2021 | EPI_ISL_13666918 | 26/7/2021 | 25 | M | Tunisia | AY.122 |
| hCoV-19/Italy/SIC-AOUP-UNIPA_86226/2021 | EPI_ISL_13666919 | 26/7/2021 | 20 | M | Tunisia | AY.122 |
| hCoV-19/Italy/SIC-AOUP-UNIPA_86230/2021 | EPI_ISL_6201738 | 26/7/2021 | 41 | M | Tunisia | AY.122 |
| hCoV-19/Italy/SIC-AOUP-UNIPA_86232/2021 | EPI_ISL_13666920 | 26/7/2021 | 22 | M | Tunisia | AY.122 |
| hCoV-19/Italy/SIC-AOUP-UNIPA_86233/2021 | EPI_ISL_13666921 | 26/7/2021 | 18 | M | Tunisia | AY.122 |

| | | | | | | |
|---|---|---|---|---|---|---|
| hCoV-19/Italy/SIC-AOUP-UNIPA_86234/2021 | EPI_ISL_13666922 | 26/7/2021 | 25 | M | Tunisia | AY.122 |
| hCoV-19/Italy/SIC-AOUP-UNIPA_86235/2021 | EPI_ISL_6201729 | 26/7/2021 | 28 | M | Tunisia | AY.122 |
| hCoV-19/Italy/SIC-AOUP-UNIPA_86243/2021 | EPI_ISL_6201725 | 26/7/2021 | 41 | M | Tunisia | AY.122 |
| hCoV-19/Italy/SIC-AOUP-UNIPA_86247/2021 | EPI_ISL_13666923 | 26/7/2021 | 32 | M | Tunisia | AY.122 |
| hCoV-19/Italy/SIC-AOUP-UNIPA_86255/2021 | EPI_ISL_13666924 | 26/7/2021 | 25 | M | Tunisia | AY.122 |
| hCoV-19/Italy/SIC-AOUP-UNIPA_86256/2021 | EPI_ISL_13666925 | 26/7/2021 | 28 | M | Tunisia | AY.122 |
| hCoV-19/Italy/SIC-AOUP-UNIPA_86265/2021 | EPI_ISL_13666926 | 26/7/2021 | 51 | M | Tunisia | AY.122 |
| hCoV-19/Italy/SIC-AOUP-UNIPA_86269/2021 | EPI_ISL_3280869 | 26/7/2021 | 24 | M | Algeria | AY.122 |
| hCoV-19/Italy/SIC-AOUP-UNIPA_86292/2021 | EPI_ISL_13666927 | 26/7/2021 | 46 | M | Tunisia | AY.122 |
| hCoV-19/Italy/SIC-AOUP-UNIPA_86299/2021 | EPI_ISL_13666928 | 26/7/2021 | 47 | M | Tunisia | AY.122 |
| hCoV-19/Italy/SIC-AOUP-UNIPA_86304/2021 | EPI_ISL_6201704 | 26/7/2021 | 17 | M | Egypt | AY.122 |
| hCoV-19/Italy/SIC-AOUP-UNIPA_86426/2021 | EPI_ISL_3280857 | 25/8/2021 | 28 | M | Bangladesh | B.1.617.2 |
| hCoV-19/Italy/SIC-AOUP-UNIPA_86428/2021 | EPI_ISL_13666929 | 25/7/2021 | 19 | M | Bangladesh | B.1.617.2 |
| hCoV-19/Italy/SIC-AOUP-UNIPA_86430/2021 | EPI_ISL_3280856 | 25/7/2021 | 27 | M | Bangladesh | B.1.617.2 |
| hCoV-19/Italy/SIC-AOUP-UNIPA_86450/2021 | EPI_ISL_13666930 | 28/7/2021 | 12 | F | Tunisia | AY.122 |
| hCoV-19/Italy/SIC-AOUP-UNIPA_86462/2021 | EPI_ISL_3280853 | 28/7/2021 | 16 | M | Guinea Conakry | B.1.617.2 |
| hCoV-19/Italy/SIC-AOUP-UNIPA_86485/2021 | EPI_ISL_13666931 | 28/7/2021 | 52 | F | Tunisia | AY.122 |
| hCoV-19/Italy/SIC-AOUP-UNIPA_86596/2021 | EPI_ISL_4050986 | 4/8/2021 | 17 | M | Bangladesh | AY.122 |
| hCoV-19/Italy/SIC-AOUP-UNIPA_86597/2021 | EPI_ISL_3915336 | 4/8/2021 | 17 | M | Tunisia | AY.122 |
| hCoV-19/Italy/SIC-AOUP-UNIPA_86608/2021 | EPI_ISL_3915339 | 29/7/2021 | 18 | M | Tunisia | AY.122 |
| hCoV-19/Italy/SIC-AOUP-UNIPA_86610/2021 | EPI_ISL_4050984 | 29/7/2021 | 18 | M | Tunisia | AY.122 |
| hCoV-19/Italy/SIC-AOUP-UNIPA_86614/2021 | EPI_ISL_4050983 | 29/7/2021 | 27 | M | Tunisia | AY.122 |

| | | | | | | |
|---|---|---|---|---|---|---|
| hCoV-19/Italy/SIC-AOUP-UNIPA_86616/2021 | EPI_ISL_4050981 | 29/7/2021 | 23 | M | Tunisia | AY.122 |
| hCoV-19/Italy/SIC-AOUP-UNIPA_86617/2021 | EPI_ISL_4050982 | 29/7/2021 | 18 | M | Tunisia | AY.122 |
| hCoV-19/Italy/SIC-AOUP-UNIPA_86618/2021 | EPI_ISL_4050979 | 29/7/2021 | 28 | M | Tunisia | AY.122 |
| hCoV-19/Italy/SIC-AOUP-UNIPA_86619/2021 | EPI_ISL_3915335 | 29/8/2021 | 28 | M | Tunisia | AY.122 |
| hCoV-19/Italy/SIC-AOUP-UNIPA_86620/2021 | EPI_ISL_4050978 | 29/7/2021 | 20 | M | Tunisia | AY.122 |
| hCoV-19/Italy/SIC-AOUP-UNIPA_86638/2021 | EPI_ISL_3915337 | 29/7/2021 | 18 | M | Tunisia | AY.122 |
| hCoV-19/Italy/SIC-AOUP-UNIPA_86639/2021 | EPI_ISL_4050980 | 30/7/2021 | 22 | M | Tunisia | AY.122 |
| hCoV-19/Italy/SIC-AOUP-UNIPA_86646/2021 | EPI_ISL_4050977 | 29/7/2021 | 21 | M | Tunisia | AY.122 |
| hCoV-19/Italy/SIC-AOUP-UNIPA_86651/2021 | EPI_ISL_3915332 | 30/7/2021 | 30 | M | Tunisia | AY.122 |
| hCoV-19/Italy/SIC-AOUP-UNIPA_86652/2021 | EPI_ISL_4050975 | 29/7/2021 | 19 | M | Tunisia | AY.122 |
| hCoV-19/Italy/SIC-AOUP-UNIPA_86654/2021 | EPI_ISL_3915331 | 30/7/2021 | 28 | M | Tunisia | AY.122 |
| hCoV-19/Italy/SIC-AOUP-UNIPA_86655/2021 | EPI_ISL_4050976 | 30/7/2021 | 28 | F | Tunisia | AY.122 |
| hCoV-19/Italy/SIC-AOUP-UNIPA_86656/2021 | EPI_ISL_3915334 | 29/7/2021 | 29 | M | Tunisia | AY.122 |
| hCoV-19/Italy/SIC-AOUP-UNIPA_86658/2021 | EPI_ISL_3915329 | 29/7/2021 | 29 | M | Tunisia | AY.122 |
| hCoV-19/Italy/SIC-AOUP-UNIPA_86659/2021 | EPI_ISL_3915330 | 29/7/2021 | 30 | M | Tunisia | AY.122 |
| hCoV-19/Italy/SIC-AOUP-UNIPA_86661/2021 | EPI_ISL_4050974 | 30/7/2021 | 0 | F | Tunisia | B.1.617.2 |
| hCoV-19/Italy/SIC-AOUP-UNIPA_86662/2021 | EPI_ISL_4050972 | 30/7/2021 | 41 | M | Tunisia | B.1.617.2 |
| hCoV-19/Italy/SIC-AOUP-UNIPA_86664/2021 | EPI_ISL_4050973 | 29/7/2021 | 26 | M | Tunisia | AY.122 |
| hCoV-19/Italy/SIC-AOUP-UNIPA_86665/2021 | EPI_ISL_3915333 | 30/7/2021 | 24 | M | Tunisia | AY.122 |
| hCoV-19/Italy/SIC-AOUP-UNIPA_86671/2021 | EPI_ISL_3915325 | 29/7/2021 | 15 | M | Tunisia | AY.122 |
| hCoV-19/Italy/SIC-AOUP-UNIPA_86676/2021 | EPI_ISL_4050970 | 30/7/2021 | 25 | M | Tunisia | AY.122 |
| hCoV-19/Italy/SIC-AOUP-UNIPA_86678/2021 | EPI_ISL_4050971 | 30/7/2021 | 34 | M | Tunisia | AY.122 |

| | | | | | | |
|---|---|---|---|---|---|---|
| hCoV-19/Italy/SIC-AOUP-UNIPA_86692/2021 | EPI_ISL_4050969 | 29/7/2021 | 26 | M | Tunisia | AY.122 |
| hCoV-19/Italy/SIC-AOUP-UNIPA_86693/2021 | EPI_ISL_3915328 | 29/7/2021 | 27 | M | Tunisia | AY.122 |
| hCoV-19/Italy/SIC-AOUP-UNIPA_86712/2021 | EPI_ISL_3915324 | 30/7/2021 | 23 | M | Tunisia | AY.122 |
| hCoV-19/Italy/SIC-AOUP-UNIPA_86714/2021 | EPI_ISL_3915323 | 29/7/2021 | 30 | M | Tunisia | AY.122 |
| hCoV-19/Italy/SIC-AOUP-UNIPA_86718/2021 | EPI_ISL_4050966 | 29/7/2021 | 32 | M | Tunisia | AY.122 |
| hCoV-19/Italy/SIC-AOUP-UNIPA_86719/2021 | EPI_ISL_3915322 | 29/7/2021 | 22 | M | Tunisia | AY.122 |
| hCoV-19/Italy/SIC-AOUP-UNIPA_86721/2021 | EPI_ISL_4050965 | 30/7/2021 | 44 | M | Tunisia | AY.122 |
| hCoV-19/Italy/SIC-AOUP-UNIPA_86722/2021 | EPI_ISL_3915327 | 30/7/2021 | 26 | M | Tunisia | AY.122 |
| hCoV-19/Italy/SIC-AOUP-UNIPA_86723/2021 | EPI_ISL_3915326 | 30/7/2021 | 41 | M | Tunisia | AY.122 |
| hCoV-19/Italy/SIC-AOUP-UNIPA_86891/2021 | EPI_ISL_13666932 | 28/7/2021 | 22 | M | Unknown | AY.122 |
| hCoV-19/Italy/SIC-AOUP-UNIPA_86976/2021 | EPI_ISL_13666933 | 5/8/2021 | 27 | F | Morocco | AY.33 |
| hCoV-19/Italy/SIC-AOUP-UNIPA_87058/2021 | EPI_ISL_4050968 | 9/8/2021 | 16 | F | Unknown | B.1.617.2 |
| hCoV-19/Italy/SIC-AOUP-UNIPA_87611/2021 | EPI_ISL_3915210 | 24/8/2021 | 25 | M | Tunisia | AY.122 |
| hCoV-19/Italy/SIC-AOUP-UNIPA_87716/2021 | EPI_ISL_3915152 | 24/8/2021 | 27 | M | Tunisia | AY.127 |
| hCoV-19/Italy/SIC-AOUP-UNIPA_87769/2021 | EPI_ISL_4050967 | 23/8/2021 | 17 | M | Bangladesh | AY.122 |
| hCoV-19/Italy/SIC-AOUP-UNIPA_87770/2021 | EPI_ISL_4050964 | 23/8/2021 | 18 | M | Bangladesh | AY.122 |
| hCoV-19/Italy/SIC-AOUP-UNIPA_87771/2021 | EPI_ISL_13666934 | 23/8/2021 | 17 | M | Unknown | AY.122 |
| hCoV-19/Italy/SIC-AOUP-UNIPA_87772/2021 | EPI_ISL_13666935 | 23/8/2021 | 17 | F | Ivory Coast | AY.122 |
| hCoV-19/Italy/SIC-AOUP-UNIPA_87775/2021 | EPI_ISL_4050963 | 23/8/2021 | 17 | M | Tunisia | AY.122 |
| hCoV-19/Italy/SIC-AOUP-UNIPA_87776/2021 | EPI_ISL_13666936 | 23/8/2021 | 17 | M | Bangladesh | AY.122 |
| hCoV-19/Italy/SIC-AOUP-UNIPA_87777/2021 | EPI_ISL_13666937 | 23/8/2021 | 17 | M | Eritrea | AY.122 |
| hCoV-19/Italy/SIC-AOUP-UNIPA_87778/2021 | EPI_ISL_13666938 | 23/8/2021 | 0 | M | Tunisia | AY.122 |

| | | | | | | |
|---|---|---|---|---|---|---|
| hCoV-19/Italy/SIC-AOUP-UNIPA_87823/2021 | EPI_ISL_13666939 | 20/8/2021 | 17 | M | Bangladesh | B.1.617.2 |
| hCoV-19/Italy/SIC-AOUP-UNIPA_88150/2021 | EPI_ISL_13666940 | 1/9/2021 | 24 | M | Tunisia | AY.122 |
| hCoV-19/Italy/SIC-AOUP-UNIPA_88155/2021 | EPI_ISL_13666941 | 1/9/2021 | 38 | M | Tunisia | AY.122 |
| hCoV-19/Italy/SIC-AOUP-UNIPA_88157/2021 | EPI_ISL_13666942 | 1/9/2021 | 35 | M | Tunisia | AY.122 |
| hCoV-19/Italy/SIC-AOUP-UNIPA_88158/2021 | EPI_ISL_13666943 | 1/9/2021 | 13 | M | Tunisia | AY.122 |
| hCoV-19/Italy/SIC-AOUP-UNIPA_88159/2021 | EPI_ISL_13666944 | 1/9/2021 | 21 | M | Tunisia | AY.122 |
| hCoV-19/Italy/SIC-AOUP-UNIPA_88160/2021 | EPI_ISL_13666945 | 1/9/2021 | 24 | M | Egypt | AY.122 |
| hCoV-19/Italy/SIC-AOUP-UNIPA_88162/2021 | EPI_ISL_13666946 | 1/9/2021 | 25 | M | Tunisia | AY.122 |
| hCoV-19/Italy/SIC-AOUP-UNIPA_88163/2021 | EPI_ISL_13666947 | 1/9/2021 | 31 | M | Tunisia | AY.36 |
| hCoV-19/Italy/SIC-AOUP-UNIPA_88164/2021 | EPI_ISL_13666948 | 1/9/2021 | 31 | M | Bangladesh | AY.122 |
| hCoV-19/Italy/SIC-AOUP-UNIPA_88167/2021 | EPI_ISL_13666949 | 1/9/2021 | 28 | M | Tunisia | AY.122 |
| hCoV-19/Italy/SIC-AOUP-UNIPA_88168/2021 | EPI_ISL_13666950 | 1/9/2021 | 20 | M | Tunisia | AY.122 |
| hCoV-19/Italy/SIC-AOUP-UNIPA_88224/2021 | EPI_ISL_13666951 | 2/9/2021 | 16 | M | Tunisia | AY.122 |
| hCoV-19/Italy/SIC-AOUP-UNIPA_88225/2021 | EPI_ISL_13666952 | 2/9/2021 | 17 | M | Tunisia | AY.122 |
| hCoV-19/Italy/SIC-AOUP-UNIPA_88226/2021 | EPI_ISL_13666953 | 2/9/2021 | 17 | M | Bangladesh | AY.122 |
| hCoV-19/Italy/SIC-AOUP-UNIPA_88227/2021 | EPI_ISL_13666954 | 2/9/2021 | 17 | M | Guinea Conakry | AY.122 |
| hCoV-19/Italy/SIC-AOUP-UNIPA_88228/2021 | EPI_ISL_13666955 | 2/9/2021 | 17 | M | Bangladesh | AY.122 |
| hCoV-19/Italy/SIC-AOUP-UNIPA_88229/2021 | EPI_ISL_13666956 | 1/9/2021 | 22 | M | Egypt | AY.127 |
| hCoV-19/Italy/SIC-AOUP-UNIPA_92000/2021 | EPI_ISL_6513122 | 4/10/2021 | 49 | F | Syria | AY.127 |
| hCoV-19/Italy/SIC-AOUP-UNIPA_92002/2021 | EPI_ISL_6513123 | 4/10/2021 | 27 | M | Egypt | AY.127 |
| hCoV-19/Italy/SIC-AOUP-UNIPA_92003/2021 | EPI_ISL_6513121 | 4/10/2021 | 37 | F | Syria | AY.127 |
| hCoV-19/Italy/SIC-AOUP-UNIPA_92008/2021 | EPI_ISL_6201266 | 4/10/2021 | 20 | M | Syria | AY.127 |

| | | | | | | |
|---|---|---|---|---|---|---|
| hCoV-19/Italy/SIC-AOUP-UNIPA_92570/2021 | EPI_ISL_6513119 | 19/10/2021 | 42 | M | Iran | AY.121 |
| hCoV-19/Italy/SIC-AOUP-UNIPA_92945/2021 | EPI_ISL_7266431 | 19/11/2021 | 36 | M | Morocco | AY.43 |
| hCoV-19/Italy/SIC-AOUP-UNIPA_96307/2022 | EPI_ISL_10335186 | 2/2/2022 | 17 | M | Bangladesh | BA.1.1 |
| hCoV-19/Italy/SIC-AOUP-UNIPA_96308/2022 | EPI_ISL_10335198 | 1/2/2022 | 21 | M | Bangladesh | BA.1.17.2 |
| hCoV-19/Italy/SIC-AOUP-UNIPA_96310/2022 | EPI_ISL_10335187 | 2/2/2022 | 32 | F | Sudan | BA.1.1 |
| hCoV-19/Italy/SIC-AOUP-UNIPA_96311/2022 | EPI_ISL_10335193 | 2/2/2022 | 18 | F | Somalia | BA.1.1 |
| hCoV-19/Italy/SIC-AOUP-UNIPA_96312/2022 | EPI_ISL_10335195 | 2/2/2022 | 31 | F | Ivory Coast | BA.1.1 |
| hCoV-19/Italy/SIC-AOUP-UNIPA_96314/2022 | EPI_ISL_10335192 | 2/2/2022 | 5 | M | Syria | BA.1.1 |
| hCoV-19/Italy/SIC-AOUP-UNIPA_96724/2022 | EPI_ISL_11149737 | 8/2/2022 | 36 | M | Bangladesh | BA.1.17.2 |
| hCoV-19/Italy/SIC-AOUP-UNIPA_96726/2022 | EPI_ISL_11149736 | 8/2/2022 | 18 | M | Bangladesh | BA.1.17.2 |
| hCoV-19/Italy/SIC-AOUP-UNIPA_96739/2022 | EPI_ISL_11149735 | 14/2/2022 | 21 | M | Bangladesh | BA.1.17.2 |
| hCoV-19/Italy/SIC-AOUP-UNIPA_96740/2022 | EPI_ISL_11149679 | 14/2/2022 | 18 | M | Tunisia | BA.1.17.2 |
| hCoV-19/Italy/SIC-AOUP-UNIPA_96742/2022 | EPI_ISL_11149734 | 14/2/2022 | 20 | M | Bangladesh | BA.1.17.2 |
| hCoV-19/Italy/SIC-AOUP-UNIPA_97045/2022 | EPI_ISL_11149725 | 21/2/2022 | 23 | M | Bangladesh | BA.2.3 |
| hCoV-19/Italy/SIC-AOUP-UNIPA_97050/2022 | EPI_ISL_11149720 | 21/2/2022 | 16 | M | Egypt | BA.1 |
| hCoV-19/Italy/SIC-AOUP-UNIPA_97051/2022 | EPI_ISL_11149719 | 21/2/2022 | 17 | F | Somalia | BA.1 |
| hCoV-19/Italy/SIC-AOUP-UNIPA_97449/2022 | EPI_ISL_12416886 | 1/2/2022 | 22 | F | Eritrea | BA.1.1 |
| hCoV-19/Italy/SIC-AOUP-UNIPA_97450/2022 | EPI_ISL_12416887 | 1/2/2022 | 28 | M | Eritrea | BA.1.1 |
| hCoV-19/Italy/SIC-AOUP-UNIPA_97454/2022 | EPI_ISL_12416885 | 2/2/2022 | 19 | M | Eritrea | BA.1.1 |
| hCoV-19/Italy/SIC-AOUP-UNIPA_97486/2022 | EPI_ISL_13637940 | 7/2/2022 | 22 | M | Bangladesh | BA.1.1 |
| hCoV-19/Italy/SIC-AOUP-UNIPA_97488/2022 | EPI_ISL_13637941 | 7/2/2022 | 30 | M | Egypt | BA.1.1 |
| hCoV-19/Italy/SIC-AOUP-UNIPA_97489/2022 | EPI_ISL_13637942 | 10/2/2022 | 24 | M | Egypt | BA.1.1 |

| | | | | | | |
|---|---|---|---|---|---|---|
| hCoV-19/Italy/SIC-AOUP-UNIPA_97490/2022 | EPI_ISL_13637943 | 10/2/2022 | 20 | F | Eritrea | BA.1.1 |
| hCoV-19/Italy/SIC-AOUP-UNIPA_97506/2022 | EPI_ISL_12416880 | 1/3/2022 | 23 | M | Ethiopia | BA.1.1 |
| hCoV-19/Italy/SIC-AOUP-UNIPA_97513/2022 | EPI_ISL_12416879 | 1/3/2022 | 31 | M | Syria | BA.1.1 |
| hCoV-19/Italy/SIC-AOUP-UNIPA_97530/2022 | EPI_ISL_12416875 | 6/3/2022 | 20 | M | Syria | BA.1 |
| hCoV-19/Italy/SIC-AOUP-UNIPA_97615/2022 | EPI_ISL_11149690 | 7/3/2022 | 32 | M | Tunisia | BA.2 |
| hCoV-19/Italy/SIC-AOUP-UNIPA_97616/2022 | EPI_ISL_11149683 | 7/3/2022 | 27 | M | Unknown | BA.2 |
| hCoV-19/Italy/SIC-AOUP-UNIPA_97650/2022 | EPI_ISL_11149665 | 7/3/2022 | 21 | M | Tunisia | BA.2 |
| hCoV-19/Italy/SIC-AOUP-UNIPA_97652/2022 | EPI_ISL_11149663 | 7/3/2022 | 25 | M | Unknown | BA.2.3 |
| hCoV-19/Italy/SIC-AOUP-UNIPA_98980/2022 | EPI_ISL_12542934 | 12/4/2022 | 21 | M | Bangladesh | BA.2 |
| hCoV-19/Italy/SIC-AOUP-UNIPA_99502/2022 | EPI_ISL_12684391 | 3/5/2022 | 44 | F | Morocco | BA.2 |
| hCoV-19/Italy/SIC-AOUP-UNIPA-59327/2021 | EPI_ISL_13531869 | 23/2/2021 | 26 | M | Egypt | A.27 |
| hCoV-19/Italy/SIC-AOUP-UNIPA-59502/2021 | EPI_ISL_1353187 | 19/2/2021 | 15 | M | Bangladesh | A.27 |
| hCoV-19/Italy/SIC-AOUP-UNIPA-82539/2021 | EPI_ISL_3915301 | 15/6/2021 | 19 | M | Syria | A.29 |

**Table S3.** Descriptions of mutations

| Location (gene) | Mutation | Count (n) | Type | Frequency (%) |
|---|---|---|---|---|
| Spike | S:D614G | 466 | Substitution | 98.7 |
| M | M:I82T | 344 | Substitution | 72.9 |
| ORF1b | ORF1b:P314L | 333 | Substitution | 70.6 |
| ORF1a | ORF1a:G3676- | 236 | Deletion | 50.0 |
| ORF1a | ORF1a:S3675- | 236 | Deletion | 50.0 |
| Spike | S:H69- | 233 | Deletion | 49.4 |
| Spike | S:V70- | 233 | Deletion | 49.4 |
| Spike | S:L452R | 224 | Substitution | 47.5 |
| ORF1a | ORF1a:F3677- | 213 | Deletion | 45.1 |
| Spike | S:T478K | 210 | Substitution | 44.5 |
| ORF1b | ORF1b:P1000L | 209 | Substitution | 44.3 |
| ORF8 | ORF8:D119- | 209 | Deletion | 44.3 |
| ORF8 | ORF8:F120- | 209 | Deletion | 44.3 |
| Spike | S:P681R | 208 | Substitution | 44.1 |
| Spike | S:Y144- | 207 | Deletion | 43.9 |
| N | N:D377Y | 206 | Substitution | 43.6 |
| N | N:D63G | 206 | Substitution | 43.6 |
| N | N:R203M | 206 | Substitution | 43.6 |
| ORF1b | ORF1b:G662S | 206 | Substitution | 43.6 |
| ORF9b | ORF9b:T60A | 206 | Substitution | 43.6 |
| Spike | S:R158G | 206 | Substitution | 43.6 |
| Spike | S:T19R | 206 | Substitution | 43.6 |
| ORF3a | ORF3a:S26L | 205 | Substitution | 43.4 |
| ORF7a | ORF7a:T120I | 205 | Substitution | 43.4 |
| ORF7a | ORF7a:V82A | 203 | Substitution | 43.0 |
| Spike | S:G142D | 201 | Substitution | 42.6 |
| Spike | S:E156- | 200 | Deletion | 42.4 |
| Spike | S:F157- | 200 | Deletion | 42.4 |
| Spike | S:E484K | 179 | Substitution | 37.9 |
| ORF1a | ORF1a:T3255I | 158 | Substitution | 33.5 |

| | | | | |
|---|---|---|---|---|
| Spike | S:D950N | 150 | Substitution | 31.8 |
| ORF1a | ORF1a:P2287S | 142 | Substitution | 30.1 |
| Spike | S:A67V | 139 | Substitution | 29.4 |
| Spike | S:Q677H | 139 | Substitution | 29.4 |
| ORF1a | ORF1a:P2046L | 130 | Substitution | 27.5 |
| ORF1a | ORF1a:T3646A | 130 | Substitution | 27.5 |
| N | N:G215C | 128 | Substitution | 27.1 |
| ORF1a | ORF1a:A1306S | 128 | Substitution | 27.1 |
| ORF1a | ORF1a:V2930L | 128 | Substitution | 27.1 |
| ORF1b | ORF1b:A1918V | 128 | Substitution | 27.1 |
| ORF7b | ORF7b:T40I | 126 | Substitution | 26.7 |
| N | N:T205I | 125 | Substitution | 26.5 |
| Spike | S:F888L | 121 | Substitution | 25.6 |
| N | N:A12G | 120 | Substitution | 25.4 |
| N | N:D3Y | 120 | Substitution | 25.4 |
| N | N:M1- | 120 | Deletion | 25.4 |
| N | N:S2M | 120 | Substitution | 25.4 |
| ORF1a | ORF1a:T2007I | 120 | Substitution | 25.4 |
| ORF6 | ORF6:F2M | 120 | Substitution | 25.4 |
| ORF6 | ORF6:M1- | 120 | Deletion | 25.4 |
| ORF9b | ORF9b:H9D | 120 | Substitution | 25.4 |
| Spike | S:Q52R | 120 | Substitution | 25.4 |
| ORF1b | ORF1b:P314F | 119 | Substitution | 25.2 |
| E | E:L21F | 115 | Substitution | 24.4 |
| ORF1a | ORF1a:K261N | 110 | Substitution | 23.3 |
| Spike | S:P681H | 110 | Substitution | 23.3 |
| ORF1a | ORF1a:A498V | 109 | Substitution | 23.1 |
| N | N:G204R | 81 | Substitution | 17.2 |
| N | N:R203K | 81 | Substitution | 17.2 |
| ORF1a | ORF1a:A3209V | 80 | Substitution | 16.9 |
| Spike | S:D1118H | 79 | Substitution | 16.7 |
| ORF1a | ORF1a:P1640L | 78 | Substitution | 16.5 |
| ORF1a | ORF1a:V3718A | 78 | Substitution | 16.5 |
| ORF7a | ORF7a:R118G | 78 | Substitution | 16.5 |

| | | | | |
|---|---|---|---|---|
| ORF1a | ORF1a:T3750I | 77 | Substitution | 16.3 |
| Spike | S:Q613H | 76 | Substitution | 16.1 |
| N | N:Q384H | 73 | Substitution | 15.5 |
| ORF1a | ORF1a:T4207I | 73 | Substitution | 15.5 |
| Spike | S:A222V | 73 | Substitution | 15.5 |
| Spike | S:R847K | 73 | Substitution | 15.5 |
| Spike | S:S477N | 61 | Substitution | 12.9 |
| ORF3a | ORF3a:G49V | 57 | Substitution | 12.1 |
| Spike | S:A243- | 56 | Deletion | 11.9 |
| Spike | S:L241- | 56 | Deletion | 11.9 |
| Spike | S:L242- | 56 | Deletion | 11.9 |
| ORF1a | ORF1a:T403I | 53 | Substitution | 11.2 |
| ORF3a | ORF3a:V13L | 53 | Substitution | 11.2 |
| N | N:A220V | 52 | Substitution | 11.0 |
| ORF1a | ORF1a:S1468F | 52 | Substitution | 11.0 |
| ORF1b | ORF1b:A1326S | 52 | Substitution | 11.0 |
| ORF3a | ORF3a:L46F | 52 | Substitution | 11.0 |
| ORF3a | ORF3a:S166L | 52 | Substitution | 11.0 |
| Spike | S:P26S | 52 | Substitution | 11.0 |
| Spike | S:T1027I | 52 | Substitution | 11.0 |
| ORF1a | ORF1a:V1991I | 51 | Substitution | 10.8 |
| ORF1b | ORF1b:A1215S | 51 | Substitution | 10.8 |
| ORF7b | ORF7b:L14- | 51 | Deletion | 10.8 |
| ORF9b | ORF9b:I5T | 51 | Substitution | 10.8 |
| Spike | S:H245Y | 51 | Substitution | 10.8 |
| Spike | S:V126A | 51 | Substitution | 10.8 |
| ORF1a | ORF1a:T1055I | 49 | Substitution | 10.4 |
| ORF1b | ORF1b:L1504F | 45 | Substitution | 9.5 |
| ORF1a | ORF1a:E269K | 40 | Substitution | 8.5 |
| ORF1b | ORF1b:P1567L | 39 | Substitution | 8.3 |
| Spike | S:N501Y | 37 | Substitution | 7.8 |
| ORF1a | ORF1a:A3969T | 35 | Substitution | 7.4 |
| Spike | S:H655Y | 35 | Substitution | 7.4 |
| Spike | S:D796Y | 34 | Substitution | 7.2 |

| | | | | |
|---|---|---|---|---|
| Spike | S:K417N | 34 | Substitution | 7.2 |
| N | N:P13L | 32 | Substitution | 6.8 |
| ORF9b | ORF9b:P10S | 32 | Substitution | 6.8 |
| E | E:T9I | 30 | Substitution | 6.4 |
| M | M:A63T | 30 | Substitution | 6.4 |
| M | M:Q19E | 30 | Substitution | 6.4 |
| N | N:E31- | 30 | Deletion | 6.4 |
| N | N:R32- | 30 | Deletion | 6.4 |
| N | N:S33- | 30 | Deletion | 6.4 |
| ORF1a | ORF1a:P3395H | 30 | Substitution | 6.4 |
| ORF1b | ORF1b:I1566V | 30 | Substitution | 6.4 |
| ORF9b | ORF9b:A29- | 30 | Deletion | 6.4 |
| ORF9b | ORF9b:E27- | 30 | Deletion | 6.4 |
| ORF9b | ORF9b:N28- | 30 | Deletion | 6.4 |
| Spike | S:G339D | 30 | Substitution | 6.4 |
| Spike | S:N440K | 30 | Substitution | 6.4 |
| Spike | S:N679K | 30 | Substitution | 6.4 |
| Spike | S:N764K | 30 | Substitution | 6.4 |
| Spike | S:N969K | 30 | Substitution | 6.4 |
| Spike | S:Q954H | 30 | Substitution | 6.4 |
| N | N:D3L | 29 | Substitution | 6.1 |
| Spike | S:S373P | 29 | Substitution | 6.1 |
| Spike | S:S375F | 29 | Substitution | 6.1 |
| Spike | S:T716I | 29 | Substitution | 6.1 |
| N | N:S235F | 28 | Substitution | 5.9 |
| ORF1a | ORF1a:A1708D | 28 | Substitution | 5.9 |
| ORF1a | ORF1a:T1001I | 28 | Substitution | 5.9 |
| ORF8 | ORF8:Q27* | 28 | Stop codon | 5.9 |
| ORF8 | ORF8:R52I | 28 | Substitution | 5.9 |
| Spike | S:A570D | 28 | Substitution | 5.9 |
| Spike | S:S982A | 28 | Substitution | 5.9 |
| ORF1a | ORF1a:I2230T | 26 | Substitution | 5.5 |
| ORF8 | ORF8:Y73C | 26 | Substitution | 5.5 |
| Spike | S:V143- | 26 | Deletion | 5.5 |

| | | | | |
|---|---|---|---|---|
| M | M:D3G | 23 | Substitution | 4.9 |
| ORF1a | ORF1a:A2710T | 23 | Substitution | 4.9 |
| ORF1a | ORF1a:I3758V | 23 | Substitution | 4.9 |
| ORF1a | ORF1a:K856R | 23 | Substitution | 4.9 |
| ORF1a | ORF1a:L2084I | 23 | Substitution | 4.9 |
| ORF1a | ORF1a:L3674- | 23 | Deletion | 4.9 |
| ORF1a | ORF1a:S2083- | 23 | Deletion | 4.9 |
| Spike | S:G446S | 23 | Substitution | 4.9 |
| Spike | S:L212I | 23 | Substitution | 4.9 |
| Spike | S:L981F | 23 | Substitution | 4.9 |
| Spike | S:N211- | 23 | Deletion | 4.9 |
| Spike | S:N856K | 23 | Substitution | 4.9 |
| Spike | S:S371L | 23 | Substitution | 4.9 |
| Spike | S:T547K | 23 | Substitution | 4.9 |
| ORF1a | ORF1a:G3278S | 21 | Substitution | 4.4 |
| ORF1a | ORF1a:T1246I | 21 | Substitution | 4.4 |
| Spike | S:G142- | 21 | Deletion | 4.4 |
| N | N:G212V | 19 | Substitution | 4.0 |
| ORF1a | ORF1a:A859V | 19 | Substitution | 4.0 |
| ORF1a | ORF1a:T4090I | 19 | Substitution | 4.0 |
| ORF8 | ORF8:P36S | 18 | Substitution | 3.8 |
| Spike | S:Y145D | 17 | Substitution | 3.6 |
| ORF1a | ORF1a:S3099L | 16 | Substitution | 3.4 |
| Spike | S:T95I | 16 | Substitution | 3.4 |
| ORF1a | ORF1a:D1639N | 15 | Substitution | 3.2 |
| ORF1a | ORF1a:D2980N | 15 | Substitution | 3.2 |
| ORF1a | ORF1a:D3222N | 15 | Substitution | 3.2 |
| ORF1a | ORF1a:E102K | 15 | Substitution | 3.2 |
| ORF1a | ORF1a:L3691S | 15 | Substitution | 3.2 |
| ORF1a | ORF1a:L4111F | 15 | Substitution | 3.2 |
| ORF1a | ORF1a:S3687L | 15 | Substitution | 3.2 |
| ORF1b | ORF1b:D1028Y | 15 | Substitution | 3.2 |
| ORF7b | ORF7b:A43S | 15 | Substitution | 3.2 |
| ORF8 | ORF8:K68* | 15 | Stop codon | 3.2 |

| | | | | |
|---|---|---|---|---|
| ORF9b | ORF9b:P3L | 15 | Substitution | 3.2 |
| Spike | S:A899S | 15 | Substitution | 3.2 |
| Spike | S:R346S | 15 | Substitution | 3.2 |
| Spike | S:S12F | 15 | Substitution | 3.2 |
| Spike | S:W152R | 15 | Substitution | 3.2 |
| ORF1a | ORF1a:G2265V | 14 | Substitution | 3.0 |
| ORF1b | ORF1b:A520V | 14 | Substitution | 3.0 |
| Spike | S:R346K | 14 | Substitution | 3.0 |
| Spike | S:L5F | 13 | Substitution | 2.8 |
| ORF1a | ORF1a:A3518V | 12 | Substitution | 2.5 |
| ORF1a | ORF1a:K3353R | 12 | Substitution | 2.5 |
| ORF1b | ORF1b:N168K | 12 | Substitution | 2.5 |
| ORF3a | ORF3a:N161Y | 12 | Substitution | 2.5 |
| ORF7b | ORF7b:L32F | 12 | Substitution | 2.5 |
| Spike | S:V1133F | 12 | Substitution | 2.5 |
| ORF1a | ORF1a:V84- | 11 | Deletion | 2.3 |
| Spike | S:S494L | 11 | Substitution | 2.3 |
| ORF1a | ORF1a:M85- | 10 | Deletion | 2.1 |
| ORF1a | ORF1a:P1640S | 10 | Substitution | 2.1 |
| Spike | S:A701V | 10 | Substitution | 2.1 |
| Spike | S:E484A | 10 | Substitution | 2.1 |
| Spike | S:Q493R | 10 | Substitution | 2.1 |
| N | N:N126K | 9 | Substitution | 1.9 |
| ORF1a | ORF1a:P309L | 9 | Substitution | 1.9 |
| ORF1b | ORF1b:A2431V | 9 | Substitution | 1.9 |
| ORF8 | ORF8:V114F | 9 | Substitution | 1.9 |
| Spike | S:A67S | 9 | Substitution | 1.9 |
| M | M:L34F | 8 | Substitution | 1.7 |
| ORF1a | ORF1a:E754K | 8 | Substitution | 1.7 |
| ORF1a | ORF1a:H2125Y | 8 | Substitution | 1.7 |
| ORF1a | ORF1a:K2589R | 8 | Substitution | 1.7 |
| ORF1a | ORF1a:V3097F | 8 | Substitution | 1.7 |
| ORF1b | ORF1b:P1570L | 8 | Substitution | 1.7 |
| ORF1b | ORF1b:T2537A | 8 | Substitution | 1.7 |

| | | | | |
|---|---|---|---|---|
| ORF3a | ORF3a:T223I | 8 | Substitution | 1.7 |
| ORF6 | ORF6:D61L | 8 | Substitution | 1.7 |
| Spike | S:A27S | 8 | Substitution | 1.7 |
| Spike | S:D138- | 8 | Deletion | 1.7 |
| Spike | S:P139- | 8 | Deletion | 1.7 |
| N | N:S413R | 7 | Substitution | 1.5 |
| ORF1a | ORF1a:G1307S | 7 | Substitution | 1.5 |
| ORF1a | ORF1a:L3027F | 7 | Substitution | 1.5 |
| ORF1a | ORF1a:L3201F | 7 | Substitution | 1.5 |
| ORF1a | ORF1a:S135R | 7 | Substitution | 1.5 |
| ORF1a | ORF1a:T842I | 7 | Substitution | 1.5 |
| ORF1b | ORF1b:K2557R | 7 | Substitution | 1.5 |
| ORF1b | ORF1b:R1315C | 7 | Substitution | 1.5 |
| ORF1b | ORF1b:T2163I | 7 | Substitution | 1.5 |
| ORF3a | ORF3a:Q57H | 7 | Substitution | 1.5 |
| ORF7a | ORF7a:P99- | 7 | Deletion | 1.5 |
| ORF7a | ORF7a:S98- | 7 | Deletion | 1.5 |
| ORF7a | ORF7a:Y97- | 7 | Deletion | 1.5 |
| Spike | S:L24- | 7 | Deletion | 1.5 |
| Spike | S:P25- | 7 | Deletion | 1.5 |
| Spike | S:P26- | 7 | Deletion | 1.5 |
| Spike | S:S371F | 7 | Substitution | 1.5 |
| Spike | S:T19I | 7 | Substitution | 1.5 |
| Spike | S:V213G | 7 | Substitution | 1.5 |
| Spike | S:Y144F | 7 | Substitution | 1.5 |
| N | N:S202N | 6 | Substitution | 1.3 |
| ORF1a | ORF1a:E1363G | 6 | Substitution | 1.3 |
| ORF1a | ORF1a:H45Y | 6 | Substitution | 1.3 |
| ORF1a | ORF1a:L4391I | 6 | Substitution | 1.3 |
| ORF1a | ORF1a:P1220S | 6 | Substitution | 1.3 |
| ORF1a | ORF1a:V1887I | 6 | Substitution | 1.3 |
| ORF1b | ORF1b:A1521V | 6 | Substitution | 1.3 |
| ORF1b | ORF1b:M1596I | 6 | Substitution | 1.3 |
| ORF1b | ORF1b:T1902I | 6 | Substitution | 1.3 |

| | | | | |
|---|---|---|---|---|
| ORF3a | ORF3a:S92L | 6 | Substitution | 1.3 |
| ORF3a | ORF3a:W131C | 6 | Substitution | 1.3 |
| ORF7a | ORF7a:E91- | 6 | Deletion | 1.3 |
| ORF7a | ORF7a:E92- | 6 | Deletion | 1.3 |
| ORF7a | ORF7a:E95- | 6 | Deletion | 1.3 |
| ORF7a | ORF7a:I88- | 6 | Deletion | 1.3 |
| ORF7a | ORF7a:L96- | 6 | Deletion | 1.3 |
| ORF7a | ORF7a:Q90- | 6 | Deletion | 1.3 |
| ORF7a | ORF7a:Q94- | 6 | Deletion | 1.3 |
| ORF7a | ORF7a:R89- | 6 | Deletion | 1.3 |
| ORF7a | ORF7a:V93- | 6 | Deletion | 1.3 |
| ORF8 | ORF8:L84S | 6 | Substitution | 1.3 |
| ORF9b | ORF9b:R32L | 6 | Substitution | 1.3 |
| Spike | S:D405N | 6 | Substitution | 1.3 |
| Spike | S:L18F | 6 | Substitution | 1.3 |
| Spike | S:Q183H | 6 | Substitution | 1.3 |
| Spike | S:R408S | 6 | Substitution | 1.3 |
| Spike | S:T376A | 6 | Substitution | 1.3 |
| ORF1a | ORF1a:A3456T | 5 | Substitution | 1.1 |
| ORF1a | ORF1a:K1512R | 5 | Substitution | 1.1 |
| ORF1a | ORF1a:M3527I | 5 | Substitution | 1.1 |
| ORF1a | ORF1a:T3090I | 5 | Substitution | 1.1 |
| ORF1a | ORF1a:V2494I | 5 | Substitution | 1.1 |
| ORF1b | ORF1b:T1637I | 5 | Substitution | 1.1 |
| ORF3a | ORF3a:K16T | 5 | Substitution | 1.1 |
| ORF3a | ORF3a:Q57R | 5 | Substitution | 1.1 |
| ORF3a | ORF3a:T221K | 5 | Substitution | 1.1 |
| ORF6 | ORF6:E59G | 5 | Substitution | 1.1 |
| ORF7a | ORF7a:L116F | 5 | Substitution | 1.1 |
| ORF7a | ORF7a:P34S | 5 | Substitution | 1.1 |
| ORF7b | ORF7b:E39* | 5 | Stop codon | 1.1 |
| Spike | S:F643L | 5 | Substitution | 1.1 |
| Spike | S:G502- | 5 | Deletion | 1.1 |
| Spike | S:G504- | 5 | Deletion | 1.1 |

| | | | | |
|---|---|---|---|---|
| Spike | S:I850L | 5 | Substitution | 1.1 |
| Spike | S:V503- | 5 | Deletion | 1.1 |
| E | E:P71L | 4 | Substitution | 0.8 |
| N | N:A414P | 4 | Substitution | 0.8 |
| ORF1a | ORF1a:A1473V | 4 | Substitution | 0.8 |
| ORF1a | ORF1a:A2855T | 4 | Substitution | 0.8 |
| ORF1a | ORF1a:A474T | 4 | Substitution | 0.8 |
| ORF1a | ORF1a:I3587T | 4 | Substitution | 0.8 |
| ORF1a | ORF1a:K1655N | 4 | Substitution | 0.8 |
| ORF1a | ORF1a:M3087I | 4 | Substitution | 0.8 |
| ORF1a | ORF1a:N1458S | 4 | Substitution | 0.8 |
| ORF1a | ORF1a:S2255F | 4 | Substitution | 0.8 |
| ORF1a | ORF1a:T265I | 4 | Substitution | 0.8 |
| ORF1b | ORF1b:A37S | 4 | Substitution | 0.8 |
| ORF1b | ORF1b:Q348H | 4 | Substitution | 0.8 |
| ORF3a | ORF3a:A110S | 4 | Substitution | 0.8 |
| ORF3a | ORF3a:M260I | 4 | Substitution | 0.8 |
| ORF3a | ORF3a:S171L | 4 | Substitution | 0.8 |
| ORF7a | ORF7a:A66V | 4 | Substitution | 0.8 |
| ORF8 | ORF8:E92K | 4 | Substitution | 0.8 |
| Spike | S:A522S | 4 | Substitution | 0.8 |
| Spike | S:D215G | 4 | Substitution | 0.8 |
| Spike | S:D80A | 4 | Substitution | 0.8 |
| Spike | S:T250I | 4 | Substitution | 0.8 |
| Spike | S:W258R | 4 | Substitution | 0.8 |
| N | N:D401Y | 3 | Substitution | 0.6 |
| ORF1a | ORF1a:A1314V | 3 | Substitution | 0.6 |
| ORF1a | ORF1a:A3924V | 3 | Substitution | 0.6 |
| ORF1a | ORF1a:D2980G | 3 | Substitution | 0.6 |
| ORF1a | ORF1a:G82- | 3 | Deletion | 0.6 |
| ORF1a | ORF1a:H83- | 3 | Deletion | 0.6 |
| ORF1a | ORF1a:L3606F | 3 | Substitution | 0.6 |
| ORF1a | ORF1a:M3655I | 3 | Substitution | 0.6 |
| ORF1a | ORF1a:N1964S | 3 | Substitution | 0.6 |

| | | | | |
|---|---|---|---|---|
| ORF1a | ORF1a:N3651S | 3 | Substitution | 0.6 |
| ORF1a | ORF1a:P286L | 3 | Substitution | 0.6 |
| ORF1a | ORF1a:P6L | 3 | Substitution | 0.6 |
| ORF1a | ORF1a:S1612L | 3 | Substitution | 0.6 |
| ORF1a | ORF1a:S40P | 3 | Substitution | 0.6 |
| ORF1a | ORF1a:T1881I | 3 | Substitution | 0.6 |
| ORF1a | ORF1a:T2906I | 3 | Substitution | 0.6 |
| ORF1a | ORF1a:T4217I | 3 | Substitution | 0.6 |
| ORF1a | ORF1a:T677I | 3 | Substitution | 0.6 |
| ORF1b | ORF1b:P1727L | 3 | Substitution | 0.6 |
| ORF1b | ORF1b:S1273L | 3 | Substitution | 0.6 |
| ORF1b | ORF1b:T1555I | 3 | Substitution | 0.6 |
| ORF1b | ORF1b:V2073L | 3 | Substitution | 0.6 |
| ORF1b | ORF1b:V767L | 3 | Substitution | 0.6 |
| ORF3a | ORF3a:N257- | 3 | Deletion | 0.6 |
| ORF3a | ORF3a:P104L | 3 | Substitution | 0.6 |
| ORF3a | ORF3a:P258- | 3 | Deletion | 0.6 |
| ORF3a | ORF3a:V50A | 3 | Substitution | 0.6 |
| ORF8 | ORF8:W45C | 3 | Substitution | 0.6 |
| Spike | S:A653V | 3 | Substitution | 0.6 |
| Spike | S:D215Y | 3 | Substitution | 0.6 |
| Spike | S:F186S | 3 | Substitution | 0.6 |
| Spike | S:G1219V | 3 | Substitution | 0.6 |
| Spike | S:L141- | 3 | Deletion | 0.6 |
| Spike | S:N501- | 3 | Deletion | 0.6 |
| Spike | S:P499- | 3 | Deletion | 0.6 |
| Spike | S:T500- | 3 | Deletion | 0.6 |
| M | M:V66L | 2 | Substitution | 0.4 |
| M | M:Y178- | 2 | Deletion | 0.4 |
| M | M:Y179- | 2 | Deletion | 0.4 |
| N | N:A156S | 2 | Substitution | 0.4 |
| N | N:A211V | 2 | Substitution | 0.4 |
| N | N:E378Q | 2 | Substitution | 0.4 |
| N | N:G18C | 2 | Substitution | 0.4 |

| | | | | |
|---|---|---|---|---|
| N | N:G19A | 2 | Substitution | 0.4 |
| N | N:P20S | 2 | Substitution | 0.4 |
| N | N:T362I | 2 | Substitution | 0.4 |
| ORF1a | ORF1a:A2909V | 2 | Substitution | 0.4 |
| ORF1a | ORF1a:A4136V | 2 | Substitution | 0.4 |
| ORF1a | ORF1a:C2239Y | 2 | Substitution | 0.4 |
| ORF1a | ORF1a:C3798- | 2 | Deletion | 0.4 |
| ORF1a | ORF1a:D1933G | 2 | Substitution | 0.4 |
| ORF1a | ORF1a:E2946D | 2 | Substitution | 0.4 |
| ORF1a | ORF1a:F143- | 2 | Deletion | 0.4 |
| ORF1a | ORF1a:F2881L | 2 | Substitution | 0.4 |
| ORF1a | ORF1a:F3794- | 2 | Deletion | 0.4 |
| ORF1a | ORF1a:F3797- | 2 | Deletion | 0.4 |
| ORF1a | ORF1a:G2207C | 2 | Substitution | 0.4 |
| ORF1a | ORF1a:G3795- | 2 | Deletion | 0.4 |
| ORF1a | ORF1a:G989V | 2 | Substitution | 0.4 |
| ORF1a | ORF1a:H1500Y | 2 | Substitution | 0.4 |
| ORF1a | ORF1a:H388R | 2 | Substitution | 0.4 |
| ORF1a | ORF1a:I1259L | 2 | Substitution | 0.4 |
| ORF1a | ORF1a:K120N | 2 | Substitution | 0.4 |
| ORF1a | ORF1a:K1348Q | 2 | Substitution | 0.4 |
| ORF1a | ORF1a:K141- | 2 | Deletion | 0.4 |
| ORF1a | ORF1a:L3667F | 2 | Substitution | 0.4 |
| ORF1a | ORF1a:L3796- | 2 | Deletion | 0.4 |
| ORF1a | ORF1a:L3799- | 2 | Deletion | 0.4 |
| ORF1a | ORF1a:L3800- | 2 | Deletion | 0.4 |
| ORF1a | ORF1a:L451F | 2 | Substitution | 0.4 |
| ORF1a | ORF1a:M3752I | 2 | Substitution | 0.4 |
| ORF1a | ORF1a:M85V | 2 | Substitution | 0.4 |
| ORF1a | ORF1a:N2596S | 2 | Substitution | 0.4 |
| ORF1a | ORF1a:N3801- | 2 | Deletion | 0.4 |
| ORF1a | ORF1a:P115S | 2 | Substitution | 0.4 |
| ORF1a | ORF1a:P3395L | 2 | Substitution | 0.4 |
| ORF1a | ORF1a:P62S | 2 | Substitution | 0.4 |

| | | | | |
|---|---|---|---|---|
| ORF1a | ORF1a:R119H | 2 | Substitution | 0.4 |
| ORF1a | ORF1a:R2115I | 2 | Substitution | 0.4 |
| ORF1a | ORF1a:R3802- | 2 | Deletion | 0.4 |
| ORF1a | ORF1a:S142- | 2 | Deletion | 0.4 |
| ORF1a | ORF1a:S2015G | 2 | Substitution | 0.4 |
| ORF1a | ORF1a:S2030L | 2 | Substitution | 0.4 |
| ORF1a | ORF1a:S443F | 2 | Substitution | 0.4 |
| ORF1a | ORF1a:S653L | 2 | Substitution | 0.4 |
| ORF1a | ORF1a:T1022I | 2 | Substitution | 0.4 |
| ORF1a | ORF1a:T1754I | 2 | Substitution | 0.4 |
| ORF1a | ORF1a:T2002M | 2 | Substitution | 0.4 |
| ORF1a | ORF1a:T3058I | 2 | Substitution | 0.4 |
| ORF1a | ORF1a:T3646I | 2 | Substitution | 0.4 |
| ORF1a | ORF1a:T4249I | 2 | Substitution | 0.4 |
| ORF1a | ORF1a:T727I | 2 | Substitution | 0.4 |
| ORF1a | ORF1a:V3718F | 2 | Substitution | 0.4 |
| ORF1a | ORF1a:V4310F | 2 | Substitution | 0.4 |
| ORF1a | ORF1a:Y3793- | 2 | Deletion | 0.4 |
| ORF1b | ORF1b:A2132V | 2 | Substitution | 0.4 |
| ORF1b | ORF1b:A2222V | 2 | Substitution | 0.4 |
| ORF1b | ORF1b:A2306V | 2 | Substitution | 0.4 |
| ORF1b | ORF1b:E1288D | 2 | Substitution | 0.4 |
| ORF1b | ORF1b:I1074V | 2 | Substitution | 0.4 |
| ORF1b | ORF1b:I97V | 2 | Substitution | 0.4 |
| ORF1b | ORF1b:K89E | 2 | Substitution | 0.4 |
| ORF1b | ORF1b:L1701F | 2 | Substitution | 0.4 |
| ORF1b | ORF1b:L2213F | 2 | Substitution | 0.4 |
| ORF1b | ORF1b:M1499I | 2 | Substitution | 0.4 |
| ORF1b | ORF1b:P2633S | 2 | Substitution | 0.4 |
| ORF1b | ORF1b:Q2635H | 2 | Substitution | 0.4 |
| ORF1b | ORF1b:R2141K | 2 | Substitution | 0.4 |
| ORF1b | ORF1b:S1985L | 2 | Substitution | 0.4 |
| ORF1b | ORF1b:S904L | 2 | Substitution | 0.4 |
| ORF1b | ORF1b:T1038I | 2 | Substitution | 0.4 |

| | | | | |
|---|---|---|---|---|
| ORF1b | ORF1b:T1137I | 2 | Substitution | 0.4 |
| ORF1b | ORF1b:T1540I | 2 | Substitution | 0.4 |
| ORF1b | ORF1b:V2178F | 2 | Substitution | 0.4 |
| ORF1b | ORF1b:V2371L | 2 | Substitution | 0.4 |
| ORF1b | ORF1b:V2691F | 2 | Substitution | 0.4 |
| ORF3a | ORF3a:A110V | 2 | Substitution | 0.4 |
| ORF3a | ORF3a:G76S | 2 | Substitution | 0.4 |
| ORF3a | ORF3a:I47V | 2 | Substitution | 0.4 |
| ORF3a | ORF3a:L140F | 2 | Substitution | 0.4 |
| ORF3a | ORF3a:T14I | 2 | Substitution | 0.4 |
| ORF3a | ORF3a:T89I | 2 | Substitution | 0.4 |
| ORF6 | ORF6:E55* | 2 | Stop codon | 0.4 |
| ORF7a | ORF7a:F59- | 2 | Deletion | 0.4 |
| ORF7a | ORF7a:G38V | 2 | Substitution | 0.4 |
| ORF7a | ORF7a:L116P | 2 | Substitution | 0.4 |
| ORF7a | ORF7a:S60- | 2 | Deletion | 0.4 |
| ORF7a | ORF7a:T61- | 2 | Deletion | 0.4 |
| ORF7a | ORF7a:V71L | 2 | Substitution | 0.4 |
| ORF7b | ORF7b:L25F | 2 | Substitution | 0.4 |
| ORF8 | ORF8:C83- | 2 | Deletion | 0.4 |
| ORF8 | ORF8:D75- | 2 | Deletion | 0.4 |
| ORF8 | ORF8:F86- | 2 | Deletion | 0.4 |
| ORF8 | ORF8:G77- | 2 | Deletion | 0.4 |
| ORF8 | ORF8:G8V | 2 | Substitution | 0.4 |
| ORF8 | ORF8:I71- | 2 | Deletion | 0.4 |
| ORF8 | ORF8:I74- | 2 | Deletion | 0.4 |
| ORF8 | ORF8:I76- | 2 | Deletion | 0.4 |
| ORF8 | ORF8:I88- | 2 | Deletion | 0.4 |
| ORF8 | ORF8:L84- | 2 | Deletion | 0.4 |
| ORF8 | ORF8:N78- | 2 | Deletion | 0.4 |
| ORF8 | ORF8:P36L | 2 | Substitution | 0.4 |
| ORF8 | ORF8:P70- | 2 | Deletion | 0.4 |
| ORF8 | ORF8:P85- | 2 | Deletion | 0.4 |
| ORF8 | ORF8:Q72- | 2 | Deletion | 0.4 |

| | | | | |
|---|---|---|---|---|
| ORF8 | ORF8:S69- | 2 | Deletion | 0.4 |
| ORF8 | ORF8:S82- | 2 | Deletion | 0.4 |
| ORF8 | ORF8:T11K | 2 | Substitution | 0.4 |
| ORF8 | ORF8:T80- | 2 | Deletion | 0.4 |
| ORF8 | ORF8:T87- | 2 | Deletion | 0.4 |
| ORF8 | ORF8:V81- | 2 | Deletion | 0.4 |
| ORF8 | ORF8:Y73- | 2 | Deletion | 0.4 |
| ORF8 | ORF8:Y79- | 2 | Deletion | 0.4 |
| ORF9b | ORF9b:A57V | 2 | Substitution | 0.4 |
| ORF9b | ORF9b:D16H | 2 | Substitution | 0.4 |
| ORF9b | ORF9b:L14F | 2 | Substitution | 0.4 |
| ORF9b | ORF9b:L64P | 2 | Substitution | 0.4 |
| ORF9b | ORF9b:R32P | 2 | Substitution | 0.4 |
| Spike | S:A1078S | 2 | Substitution | 0.4 |
| Spike | S:A672V | 2 | Substitution | 0.4 |
| Spike | S:A688V | 2 | Substitution | 0.4 |
| Spike | S:F140- | 2 | Deletion | 0.4 |
| Spike | S:F157L | 2 | Substitution | 0.4 |
| Spike | S:F186L | 2 | Substitution | 0.4 |
| Spike | S:L821V | 2 | Substitution | 0.4 |
| Spike | S:P812S | 2 | Substitution | 0.4 |
| Spike | S:P9L | 2 | Substitution | 0.4 |
| Spike | S:V367F | 2 | Substitution | 0.4 |
| Spike | S:W64R | 2 | Substitution | 0.4 |
| E | E:T11M | 1 | Substitution | 0.2 |
| M | M:I82S | 1 | Substitution | 0.2 |
| N | N:A152S | 1 | Substitution | 0.2 |
| N | N:A208- | 1 | Deletion | 0.2 |
| N | N:A35T | 1 | Substitution | 0.2 |
| N | N:A376T | 1 | Substitution | 0.2 |
| N | N:A414S | 1 | Substitution | 0.2 |
| N | N:A419T | 1 | Substitution | 0.2 |
| N | N:D128Y | 1 | Substitution | 0.2 |
| N | N:D22Y | 1 | Substitution | 0.2 |

| | | | | |
|---|---|---|---|---|
| N | N:G238C | 1 | Substitution | 0.2 |
| N | N:G243C | 1 | Substitution | 0.2 |
| N | N:G25S | 1 | Substitution | 0.2 |
| N | N:K361R | 1 | Substitution | 0.2 |
| N | N:L139F | 1 | Substitution | 0.2 |
| N | N:L221F | 1 | Substitution | 0.2 |
| N | N:M234I | 1 | Substitution | 0.2 |
| N | N:P142S | 1 | Substitution | 0.2 |
| N | N:P151L | 1 | Substitution | 0.2 |
| N | N:P326L | 1 | Substitution | 0.2 |
| N | N:P80R | 1 | Substitution | 0.2 |
| N | N:Q70R | 1 | Substitution | 0.2 |
| N | N:Q9L | 1 | Substitution | 0.2 |
| N | N:R209G | 1 | Substitution | 0.2 |
| N | N:S187L | 1 | Substitution | 0.2 |
| N | N:T135I | 1 | Substitution | 0.2 |
| N | N:T245I | 1 | Substitution | 0.2 |
| ORF1a | ORF1a:A1092V | 1 | Substitution | 0.2 |
| ORF1a | ORF1a:A1679V | 1 | Substitution | 0.2 |
| ORF1a | ORF1a:A3070T | 1 | Substitution | 0.2 |
| ORF1a | ORF1a:A3392V | 1 | Substitution | 0.2 |
| ORF1a | ORF1a:A3456- | 1 | Deletion | 0.2 |
| ORF1a | ORF1a:A3457- | 1 | Deletion | 0.2 |
| ORF1a | ORF1a:A3623S | 1 | Substitution | 0.2 |
| ORF1a | ORF1a:A4016V | 1 | Substitution | 0.2 |
| ORF1a | ORF1a:A903V | 1 | Substitution | 0.2 |
| ORF1a | ORF1a:C3790- | 1 | Deletion | 0.2 |
| ORF1a | ORF1a:C3790F | 1 | Substitution | 0.2 |
| ORF1a | ORF1a:C3792- | 1 | Deletion | 0.2 |
| ORF1a | ORF1a:D3022N | 1 | Substitution | 0.2 |
| ORF1a | ORF1a:D335N | 1 | Substitution | 0.2 |
| ORF1a | ORF1a:D3508G | 1 | Substitution | 0.2 |
| ORF1a | ORF1a:D4165A | 1 | Substitution | 0.2 |
| ORF1a | ORF1a:D953Y | 1 | Substitution | 0.2 |

| | | | | |
|---|---|---|---|---|
| ORF1a | ORF1a:E1192K | 1 | Substitution | 0.2 |
| ORF1a | ORF1a:E1209D | 1 | Substitution | 0.2 |
| ORF1a | ORF1a:E1384K | 1 | Substitution | 0.2 |
| ORF1a | ORF1a:E2050K | 1 | Substitution | 0.2 |
| ORF1a | ORF1a:E37A | 1 | Substitution | 0.2 |
| ORF1a | ORF1a:E87K | 1 | Substitution | 0.2 |
| ORF1a | ORF1a:G1125C | 1 | Substitution | 0.2 |
| ORF1a | ORF1a:G3458T | 1 | Substitution | 0.2 |
| ORF1a | ORF1a:G94V | 1 | Substitution | 0.2 |
| ORF1a | ORF1a:H1113Y | 1 | Substitution | 0.2 |
| ORF1a | ORF1a:H2799Y | 1 | Substitution | 0.2 |
| ORF1a | ORF1a:I114V | 1 | Substitution | 0.2 |
| ORF1a | ORF1a:I1398V | 1 | Substitution | 0.2 |
| ORF1a | ORF1a:I1551V | 1 | Substitution | 0.2 |
| ORF1a | ORF1a:I3927T | 1 | Substitution | 0.2 |
| ORF1a | ORF1a:I4098T | 1 | Substitution | 0.2 |
| ORF1a | ORF1a:I476V | 1 | Substitution | 0.2 |
| ORF1a | ORF1a:K1247N | 1 | Substitution | 0.2 |
| ORF1a | ORF1a:K1407N | 1 | Substitution | 0.2 |
| ORF1a | ORF1a:K1763N | 1 | Substitution | 0.2 |
| ORF1a | ORF1a:K1795Q | 1 | Substitution | 0.2 |
| ORF1a | ORF1a:K1949E | 1 | Substitution | 0.2 |
| ORF1a | ORF1a:K3363R | 1 | Substitution | 0.2 |
| ORF1a | ORF1a:K3861R | 1 | Substitution | 0.2 |
| ORF1a | ORF1a:K798N | 1 | Substitution | 0.2 |
| ORF1a | ORF1a:L1270F | 1 | Substitution | 0.2 |
| ORF1a | ORF1a:L2062F | 1 | Substitution | 0.2 |
| ORF1a | ORF1a:L2250S | 1 | Substitution | 0.2 |
| ORF1a | ORF1a:L3201I | 1 | Substitution | 0.2 |
| ORF1a | ORF1a:M1769I | 1 | Substitution | 0.2 |
| ORF1a | ORF1a:M2259T | 1 | Substitution | 0.2 |
| ORF1a | ORF1a:M3621I | 1 | Substitution | 0.2 |
| ORF1a | ORF1a:M3934I | 1 | Substitution | 0.2 |
| ORF1a | ORF1a:N1922S | 1 | Substitution | 0.2 |

| | | | | |
|---|---|---|---|---|
| ORF1a | ORF1a:N3537S | 1 | Substitution | 0.2 |
| ORF1a | ORF1a:P1786S | 1 | Substitution | 0.2 |
| ORF1a | ORF1a:P1862L | 1 | Substitution | 0.2 |
| ORF1a | ORF1a:P1921L | 1 | Substitution | 0.2 |
| ORF1a | ORF1a:P197S | 1 | Substitution | 0.2 |
| ORF1a | ORF1a:P309Q | 1 | Substitution | 0.2 |
| ORF1a | ORF1a:P3395S | 1 | Substitution | 0.2 |
| ORF1a | ORF1a:P4120T | 1 | Substitution | 0.2 |
| ORF1a | ORF1a:P4197S | 1 | Substitution | 0.2 |
| ORF1a | ORF1a:P892S | 1 | Substitution | 0.2 |
| ORF1a | ORF1a:Q1140L | 1 | Substitution | 0.2 |
| ORF1a | ORF1a:Q2574E | 1 | Substitution | 0.2 |
| ORF1a | ORF1a:Q985K | 1 | Substitution | 0.2 |
| ORF1a | ORF1a:R1467K | 1 | Substitution | 0.2 |
| ORF1a | ORF1a:R402C | 1 | Substitution | 0.2 |
| ORF1a | ORF1a:S1188L | 1 | Substitution | 0.2 |
| ORF1a | ORF1a:S1272G | 1 | Substitution | 0.2 |
| ORF1a | ORF1a:S2114F | 1 | Substitution | 0.2 |
| ORF1a | ORF1a:S2285F | 1 | Substitution | 0.2 |
| ORF1a | ORF1a:S2797Y | 1 | Substitution | 0.2 |
| ORF1a | ORF1a:S2981F | 1 | Substitution | 0.2 |
| ORF1a | ORF1a:S3983F | 1 | Substitution | 0.2 |
| ORF1a | ORF1a:S838N | 1 | Substitution | 0.2 |
| ORF1a | ORF1a:T1000I | 1 | Substitution | 0.2 |
| ORF1a | ORF1a:T1246P | 1 | Substitution | 0.2 |
| ORF1a | ORF1a:T1395A | 1 | Substitution | 0.2 |
| ORF1a | ORF1a:T1597I | 1 | Substitution | 0.2 |
| ORF1a | ORF1a:T1840I | 1 | Substitution | 0.2 |
| ORF1a | ORF1a:T2137S | 1 | Substitution | 0.2 |
| ORF1a | ORF1a:T2153I | 1 | Substitution | 0.2 |
| ORF1a | ORF1a:T2936I | 1 | Substitution | 0.2 |
| ORF1a | ORF1a:T3284I | 1 | Substitution | 0.2 |
| ORF1a | ORF1a:T329I | 1 | Substitution | 0.2 |
| ORF1a | ORF1a:T3791- | 1 | Deletion | 0.2 |

| | | | | |
|---|---|---|---|---|
| ORF1a | ORF1a:T4035I | 1 | Substitution | 0.2 |
| ORF1a | ORF1a:T4174A | 1 | Substitution | 0.2 |
| ORF1a | ORF1a:T4265I | 1 | Substitution | 0.2 |
| ORF1a | ORF1a:T4304I | 1 | Substitution | 0.2 |
| ORF1a | ORF1a:T945A | 1 | Substitution | 0.2 |
| ORF1a | ORF1a:V1177I | 1 | Substitution | 0.2 |
| ORF1a | ORF1a:V1236F | 1 | Substitution | 0.2 |
| ORF1a | ORF1a:V2816A | 1 | Substitution | 0.2 |
| ORF1a | ORF1a:V627F | 1 | Substitution | 0.2 |
| ORF1a | ORF1a:V86- | 1 | Deletion | 0.2 |
| ORF1a | ORF1a:V86F | 1 | Substitution | 0.2 |
| ORF1a | ORF1a:Y1183C | 1 | Substitution | 0.2 |
| ORF1b | ORF1b:A176S | 1 | Substitution | 0.2 |
| ORF1b | ORF1b:A2131V | 1 | Substitution | 0.2 |
| ORF1b | ORF1b:A2143V | 1 | Substitution | 0.2 |
| ORF1b | ORF1b:A2188V | 1 | Substitution | 0.2 |
| ORF1b | ORF1b:A302S | 1 | Substitution | 0.2 |
| ORF1b | ORF1b:A397V | 1 | Substitution | 0.2 |
| ORF1b | ORF1b:A647S | 1 | Substitution | 0.2 |
| ORF1b | ORF1b:C721R | 1 | Substitution | 0.2 |
| ORF1b | ORF1b:D295Y | 1 | Substitution | 0.2 |
| ORF1b | ORF1b:E1184D | 1 | Substitution | 0.2 |
| ORF1b | ORF1b:E1264D | 1 | Substitution | 0.2 |
| ORF1b | ORF1b:F210L | 1 | Substitution | 0.2 |
| ORF1b | ORF1b:G1772V | 1 | Substitution | 0.2 |
| ORF1b | ORF1b:H1087Y | 1 | Substitution | 0.2 |
| ORF1b | ORF1b:I1181T | 1 | Substitution | 0.2 |
| ORF1b | ORF1b:I1987V | 1 | Substitution | 0.2 |
| ORF1b | ORF1b:I1998V | 1 | Substitution | 0.2 |
| ORF1b | ORF1b:I2147V | 1 | Substitution | 0.2 |
| ORF1b | ORF1b:I2566V | 1 | Substitution | 0.2 |
| ORF1b | ORF1b:I2687V | 1 | Substitution | 0.2 |
| ORF1b | ORF1b:K1141R | 1 | Substitution | 0.2 |
| ORF1b | ORF1b:K2579N | 1 | Substitution | 0.2 |

| | | | | |
|---|---|---|---|---|
| ORF1b | ORF1b:L829I | 1 | Substitution | 0.2 |
| ORF1b | ORF1b:N2328D | 1 | Substitution | 0.2 |
| ORF1b | ORF1b:P1427S | 1 | Substitution | 0.2 |
| ORF1b | ORF1b:P1763L | 1 | Substitution | 0.2 |
| ORF1b | ORF1b:P1975S | 1 | Substitution | 0.2 |
| ORF1b | ORF1b:P2116S | 1 | Substitution | 0.2 |
| ORF1b | ORF1b:P218L | 1 | Substitution | 0.2 |
| ORF1b | ORF1b:P2256S | 1 | Substitution | 0.2 |
| ORF1b | ORF1b:P821S | 1 | Substitution | 0.2 |
| ORF1b | ORF1b:Q1509L | 1 | Substitution | 0.2 |
| ORF1b | ORF1b:Q2070H | 1 | Substitution | 0.2 |
| ORF1b | ORF1b:R2613C | 1 | Substitution | 0.2 |
| ORF1b | ORF1b:R2613H | 1 | Substitution | 0.2 |
| ORF1b | ORF1b:R574G | 1 | Substitution | 0.2 |
| ORF1b | ORF1b:S2689G | 1 | Substitution | 0.2 |
| ORF1b | ORF1b:T1774I | 1 | Substitution | 0.2 |
| ORF1b | ORF1b:T2537I | 1 | Substitution | 0.2 |
| ORF1b | ORF1b:V1375L | 1 | Substitution | 0.2 |
| ORF1b | ORF1b:V1538L | 1 | Substitution | 0.2 |
| ORF1b | ORF1b:V1691I | 1 | Substitution | 0.2 |
| ORF1b | ORF1b:V2368F | 1 | Substitution | 0.2 |
| ORF1b | ORF1b:V2371M | 1 | Substitution | 0.2 |
| ORF1b | ORF1b:V248I | 1 | Substitution | 0.2 |
| ORF1b | ORF1b:V389I | 1 | Substitution | 0.2 |
| ORF3a | ORF3a:A23- | 1 | Deletion | 0.2 |
| ORF3a | ORF3a:A99V | 1 | Substitution | 0.2 |
| ORF3a | ORF3a:D155Y | 1 | Substitution | 0.2 |
| ORF3a | ORF3a:D22- | 1 | Deletion | 0.2 |
| ORF3a | ORF3a:D22Y | 1 | Substitution | 0.2 |
| ORF3a | ORF3a:D238Y | 1 | Substitution | 0.2 |
| ORF3a | ORF3a:D27- | 1 | Deletion | 0.2 |
| ORF3a | ORF3a:E19- | 1 | Deletion | 0.2 |
| ORF3a | ORF3a:F28- | 1 | Deletion | 0.2 |
| ORF3a | ORF3a:G172C | 1 | Substitution | 0.2 |

| | | | | |
|---|---|---|---|---|
| ORF3a | ORF3a:G254R | 1 | Substitution | 0.2 |
| ORF3a | ORF3a:I20- | 1 | Deletion | 0.2 |
| ORF3a | ORF3a:K21- | 1 | Deletion | 0.2 |
| ORF3a | ORF3a:L111I | 1 | Substitution | 0.2 |
| ORF3a | ORF3a:L85F | 1 | Substitution | 0.2 |
| ORF3a | ORF3a:P25- | 1 | Deletion | 0.2 |
| ORF3a | ORF3a:P25S | 1 | Substitution | 0.2 |
| ORF3a | ORF3a:R134C | 1 | Substitution | 0.2 |
| ORF3a | ORF3a:R68I | 1 | Substitution | 0.2 |
| ORF3a | ORF3a:S216P | 1 | Substitution | 0.2 |
| ORF3a | ORF3a:S253P | 1 | Substitution | 0.2 |
| ORF3a | ORF3a:S26- | 1 | Deletion | 0.2 |
| ORF3a | ORF3a:S74F | 1 | Substitution | 0.2 |
| ORF3a | ORF3a:T24- | 1 | Deletion | 0.2 |
| ORF3a | ORF3a:V112F | 1 | Substitution | 0.2 |
| ORF3a | ORF3a:V197L | 1 | Substitution | 0.2 |
| ORF3a | ORF3a:V202L | 1 | Substitution | 0.2 |
| ORF3a | ORF3a:W45L | 1 | Substitution | 0.2 |
| ORF6 | ORF6:H3Y | 1 | Substitution | 0.2 |
| ORF6 | ORF6:N34S | 1 | Substitution | 0.2 |
| ORF6 | ORF6:T10I | 1 | Substitution | 0.2 |
| ORF7a | ORF7a:A55- | 1 | Deletion | 0.2 |
| ORF7a | ORF7a:A8T | 1 | Substitution | 0.2 |
| ORF7a | ORF7a:C58- | 1 | Deletion | 0.2 |
| ORF7a | ORF7a:E95* | 1 | Stop codon | 0.2 |
| ORF7a | ORF7a:I100- | 1 | Deletion | 0.2 |
| ORF7a | ORF7a:I103V | 1 | Substitution | 0.2 |
| ORF7a | ORF7a:I10V | 1 | Substitution | 0.2 |
| ORF7a | ORF7a:L56- | 1 | Deletion | 0.2 |
| ORF7a | ORF7a:P34L | 1 | Substitution | 0.2 |
| ORF7a | ORF7a:P45L | 1 | Substitution | 0.2 |
| ORF7a | ORF7a:P99S | 1 | Substitution | 0.2 |
| ORF7a | ORF7a:Q62- | 1 | Deletion | 0.2 |
| ORF7a | ORF7a:T115S | 1 | Substitution | 0.2 |

| | | | | |
|---|---|---|---|---|
| ORF7a | ORF7a:T57- | 1 | Deletion | 0.2 |
| ORF7a | ORF7a:T61I | 1 | Substitution | 0.2 |
| ORF7b | ORF7b:*44- | 1 | Deletion, stop codon | 0.2 |
| ORF7b | ORF7b:C41F | 1 | Substitution | 0.2 |
| ORF7b | ORF7b:C41S | 1 | Substitution | 0.2 |
| ORF7b | ORF7b:I2N | 1 | Substitution | 0.2 |
| ORF7b | ORF7b:T40L | 1 | Substitution | 0.2 |
| ORF8 | ORF8:A65S | 1 | Substitution | 0.2 |
| ORF8 | ORF8:E106* | 1 | Stop codon | 0.2 |
| ORF8 | ORF8:G66C | 1 | Substitution | 0.2 |
| ORF8 | ORF8:K2- | 1 | Deletion | 0.2 |
| ORF8 | ORF8:M1- | 1 | Deletion | 0.2 |
| ORF8 | ORF8:Q91H | 1 | Substitution | 0.2 |
| ORF8 | ORF8:S54L | 1 | Substitution | 0.2 |
| ORF8 | ORF8:S67F | 1 | Substitution | 0.2 |
| ORF8 | ORF8:T87I | 1 | Substitution | 0.2 |
| ORF8 | ORF8:V62L | 1 | Substitution | 0.2 |
| ORF9b | ORF9b:G38D | 1 | Substitution | 0.2 |
| ORF9b | ORF9b:K67E | 1 | Substitution | 0.2 |
| ORF9b | ORF9b:Q18H | 1 | Substitution | 0.2 |
| ORF9b | ORF9b:Q77E | 1 | Substitution | 0.2 |
| ORF9b | ORF9b:S50L | 1 | Substitution | 0.2 |
| ORF9b | ORF9b:S6C | 1 | Substitution | 0.2 |
| Spike | S:A435- | 1 | Deletion | 0.2 |
| Spike | S:A771S | 1 | Substitution | 0.2 |
| Spike | S:A831V | 1 | Substitution | 0.2 |
| Spike | S:C432- | 1 | Deletion | 0.2 |
| Spike | S:D1153Y | 1 | Substitution | 0.2 |
| Spike | S:D138Y | 1 | Substitution | 0.2 |
| Spike | S:D178G | 1 | Substitution | 0.2 |
| Spike | S:D53- | 1 | Deletion | 0.2 |
| Spike | S:D627E | 1 | Substitution | 0.2 |
| Spike | S:D796H | 1 | Substitution | 0.2 |
| Spike | S:E1195D | 1 | Substitution | 0.2 |

| | | | | |
|---|---|---|---|---|
| Spike | S:F220L | 1 | Substitution | 0.2 |
| Spike | S:F429- | 1 | Deletion | 0.2 |
| Spike | S:F43- | 1 | Deletion | 0.2 |
| Spike | S:F497- | 1 | Deletion | 0.2 |
| Spike | S:F55- | 1 | Deletion | 0.2 |
| Spike | S:F565L | 1 | Substitution | 0.2 |
| Spike | S:F58- | 1 | Deletion | 0.2 |
| Spike | S:F59- | 1 | Deletion | 0.2 |
| Spike | S:F797C | 1 | Substitution | 0.2 |
| Spike | S:F79L | 1 | Substitution | 0.2 |
| Spike | S:F86- | 1 | Deletion | 0.2 |
| Spike | S:G1124C | 1 | Substitution | 0.2 |
| Spike | S:G431- | 1 | Deletion | 0.2 |
| Spike | S:G446V | 1 | Substitution | 0.2 |
| Spike | S:G496- | 1 | Deletion | 0.2 |
| Spike | S:G496S | 1 | Substitution | 0.2 |
| Spike | S:G832R | 1 | Substitution | 0.2 |
| Spike | S:H49- | 1 | Deletion | 0.2 |
| Spike | S:I434- | 1 | Deletion | 0.2 |
| Spike | S:K1191N | 1 | Substitution | 0.2 |
| Spike | S:K417T | 1 | Substitution | 0.2 |
| Spike | S:L176F | 1 | Substitution | 0.2 |
| Spike | S:L212E | 1 | Substitution | 0.2 |
| Spike | S:L48- | 1 | Deletion | 0.2 |
| Spike | S:L54- | 1 | Deletion | 0.2 |
| Spike | S:L56- | 1 | Deletion | 0.2 |
| Spike | S:M1237I | 1 | Substitution | 0.2 |
| Spike | S:N148Y | 1 | Substitution | 0.2 |
| Spike | S:N211P | 1 | Substitution | 0.2 |
| Spike | S:N437- | 1 | Deletion | 0.2 |
| Spike | S:N450K | 1 | Substitution | 0.2 |
| Spike | S:N61- | 1 | Deletion | 0.2 |
| Spike | S:P1263L | 1 | Substitution | 0.2 |
| Spike | S:P57- | 1 | Deletion | 0.2 |

| | | | | |
|---|---|---|---|---|
| Spike | S:P85- | 1 | Deletion | 0.2 |
| Spike | S:Q1208H | 1 | Substitution | 0.2 |
| Spike | S:Q414K | 1 | Substitution | 0.2 |
| Spike | S:Q498- | 1 | Deletion | 0.2 |
| Spike | S:Q52- | 1 | Deletion | 0.2 |
| Spike | S:Q607L | 1 | Substitution | 0.2 |
| Spike | S:Q675H | 1 | Substitution | 0.2 |
| Spike | S:R190S | 1 | Substitution | 0.2 |
| Spike | S:R214E | 1 | Substitution | 0.2 |
| Spike | S:R34L | 1 | Substitution | 0.2 |
| Spike | S:R44- | 1 | Deletion | 0.2 |
| Spike | S:S45- | 1 | Deletion | 0.2 |
| Spike | S:S46- | 1 | Deletion | 0.2 |
| Spike | S:S494- | 1 | Deletion | 0.2 |
| Spike | S:S50- | 1 | Deletion | 0.2 |
| Spike | S:S60- | 1 | Deletion | 0.2 |
| Spike | S:S813N | 1 | Substitution | 0.2 |
| Spike | S:S939F | 1 | Substitution | 0.2 |
| Spike | S:T20N | 1 | Substitution | 0.2 |
| Spike | S:T299I | 1 | Substitution | 0.2 |
| Spike | S:T29A | 1 | Substitution | 0.2 |
| Spike | S:T29I | 1 | Substitution | 0.2 |
| Spike | S:T430- | 1 | Deletion | 0.2 |
| Spike | S:T51- | 1 | Deletion | 0.2 |
| Spike | S:V1104L | 1 | Substitution | 0.2 |
| Spike | S:V1176F | 1 | Substitution | 0.2 |
| Spike | S:V1228L | 1 | Substitution | 0.2 |
| Spike | S:V143F | 1 | Substitution | 0.2 |
| Spike | S:V213A | 1 | Substitution | 0.2 |
| Spike | S:V213P | 1 | Substitution | 0.2 |
| Spike | S:V433- | 1 | Deletion | 0.2 |
| Spike | S:V47- | 1 | Deletion | 0.2 |
| Spike | S:W436- | 1 | Deletion | 0.2 |
| Spike | S:Y144V | 1 | Substitution | 0.2 |

| | | | | |
|---|---|---|---|---|
| Spike | S:Y449H | 1 | Substitution | 0.2 |
| Spike | S:Y495- | 1 | Deletion | 0.2 |
